# Two divergent haplogroups of a sacsin-like gene in *Acropora corals*

**DOI:** 10.1038/s41598-021-02386-w

**Published:** 2021-11-26

**Authors:** Shiho Takahashi-Kariyazono, Yohey Terai

**Affiliations:** Department of Evolutionary Studies of Biosystems, SOKENDAI (The Graduate University for Advanced Studies), Shonan Village, Hayama, 240-0193 Japan

**Keywords:** Marine biology, Molecular evolution, Evolutionary biology

## Abstract

Reef-building corals are declining due to environmental changes. Sacsin is a member of the heat shock proteins and has been reported as a candidate protein associated with the stress response in *Acropora* corals. Recently, high nucleotide diversity and the persistence of two divergent haplogroups of sacsin-like genes in *Acropora millepora* have been reported. While it was not clear when the two haplogroups have split and whether the haplogroups have persisted in only *A. millepora* or the other lineages in the genus *Acropora*. In this study, we analyzed a genomic region containing a sacsin-like gene from *Acropora* and *Montipora* species. Higher nucleotide diversity in the sacsin-like gene compared with that of surrounding regions was also observed in *A. digitifera*. This nucleotide diversity is derived from two divergent haplogroups of a sacsin-like gene, which are present in at least three *Acropora* species. The origin of these two haplogroups can be traced back before the divergence of *Acropora* and *Montipora* (119 Ma). Although the link between exceptionally high genetic variation in sacsin-like genes and functional differences in sacsin-like proteins is not clear, the divergent haplogroups may respond differently to envionmental stressors and serve in the adaptive phsiological ecology of these keystone species.

## Introduction

Reef-building corals (Scleractinia) are declining due to heat stress caused by global warming^1^. When corals are under heat stress, the symbiotic relationship with symbiotic dinoflagellates (family Symbiodiniaceae) breaks down and result in coral death^1,2^ *Acropora* is one of the most abundant genera in the Indo-Pacific region, and includes more than 100 species^3,4^. *Acropora* species are vulnerable to heat stress; for example, almost all colonies of *Acropora* except for juveniles at the study site in Okinawa, Japan, could not survive the 1998 mass-bleaching event^5^.

Knowledge of what biological responses occur in corals under environmental stress can help us to predict what will happen to corals in the future as a result of environmental changes. Information on molecules associated with biological responses to environmental stress is important for uncovering the biological mechanisms of stress responses in corals.

Heat shock proteins (HPS) are molecular chaperones that protect cells by stabilizing the folding of damaged proteins^6^. Sacsin belongs to the HSP40 (40 kDa molecular mass) family that acts as the HSP70 (70 kDa molecular mass) family of co-chaperones^7,8^ whereby some of Hsp40s may recruit an Hsp70 to a specific set of substrates^9^.

In corals, several cases of the stress responses of sacsin have been reported. Under experimentally elevated temperatures, sacsin was present in *Pocillopora acuta,* although it was absent in the controls^10^. A sacsin-like gene was highly expressed at branches compared with branch tips in *A. palmata*, may be a response to high concentrations of reactive oxygen species (ROS) in the symbiont-rich regions of branches^11^. Exposure to an immunogen (polyinosinic:polycytidylic acid) affects sacsin-like gene expression in *A. millepora*^12^.

Genes related to local adaptation of corals may have been maintained over long periods of time because dispersing larvae may experience strong selection in each generation when they settle in a heterogenous environment with different stress factors^13^. Indeed, a genome scan for local adaptation by balancing selection in *A. millepora* pointed to a region containing a sacsin-like gene (annotated as Amillepora11972)^13^. The origin of two divergent haplogroups of this sacsin-like gene was expected to predate the divergence of *A. digitifera* and *A. tenuis,* because sacsin-like genes in *A. digitifera* and *A. tenuis* genome assembly were related to a single haplogroup^13^*.* However, since only sequences belonging to one haplogroup each were analyzed from these two species (*A. digitifera* and *A. tenuis*), it was not clear whether the haplogroups of the sacsin-like gene have persisted in only *A. millepora* or also in other lineages in the genus *Acropora*. According to heat shock protein function and balancing selection, two divergent haplogroups in a sacsin-like gene may also be associated with heat response in natural populations of *A. millepora*^13^. If a sacsin-like gene has played a role in local adaptation, the two divergent haplogroups may have persisted in lineages of *Acropora* by balancing selection, because the larvae of other members of this genus also disperse and settle on different environments with favorable haplogroups.

In this study, we analyzed nucleotide sequence of the sacsin-like gene from two species from the genus *Acropora* (*A. digitifera* and *A. tenuis*)*,* and two species from the genus *Montipora* (*M. informis* and *M. aequituberculata*) which is a sister clade of *Acropora*, and a genomic region including a sacsin-like gene from *A. digitifera.* We revealed that two haplogroups of the sacsin-like gene exist in *Acropora* and these haplogroups have persisted before the divergence of the genera *Acropora* and *Montipora* (119 Ma^14^)*.*

## Methods

### Verification of coding sequence (CDS) of a sacsin-like gene

The genomic assembly version 2.01 of *A. millepora*^13^ was downloaded from the web site (https://przeworskilab.com/acropora-millepora-genome/). RNA-seq reads of one *A. millepora* (accession: SRR2086157) were mapped (similarity fraction > 0.8, length fraction > 0.9) to the genome assembly using CLC Genomics Workbench (https://www.qiagenbioinformatics.com/). Mapping results were checked manualy and the coding sequence (CDS) of the sacsin-like gene was determined. To identify conserved domains in the sacsin-like gene, the CDS was translated to amino acids and used as the query for an homology search by Phyre2 (Protein Homology/analogY Recognition Engine V 2.0)^15^.

### Sliding window analysis of nucleotide diversity (Pi) among A. digitifera in a genomic region containing the sacsin-like gene

The publicly available genomic DNA reads of *A. digitifera* collected from the southern Ryukyu Archipelago located in southwestern Japan were used^16^. In total, 33 individuals with read mapping coverage over 9.5 × were selected. The accession numbers for genomic DNA reads are shown in Table S1. Sequence reads were mapped to the *A. millepora* genome assembly version 2.01 using CLC Genomics Workbench with the same mapping conditions mentioned above. Consensus sequences of a region containing the sacsin-like gene (chr7: 13,304,000–13,335,000) were extracted from each of all individuals using CLC Genomics Workbench with the following condition: minimum nucleotide count = 10; Noise threshold = 0.3. To remove a repetitive sequence region (chr7: 13,313,503–13,318,235) from the following analysis, these consensus sequences were divided into two parts, one upstream and one downstream, of the repetitive sequence. The consensus sequences were divided into alleles by a perl script (see Supplementary Material: 2 Description of the script) to import the sequences into DnaSP v5^17^. Nucleotide diversity (Pi) was calculated with sliding window analysis (window length = 100 bp and step size = 25 bp) using DnaSP v5^17^.

### Identification and cloning of sacsin-like gene sequences

Genomic DNA was extracted from three *A. digitifera* and two *A. tenuis* individuals from our previous study^18,19^. Genomic DNA from single colonies of *M. informis* and *M. aequituberculata* were provided by Dr. Masayuki Hatta. Genomic DNA from each individual was used for templates of polymerase chain reaction (PCR).

A locus (chr 7: 13,325,506–13,326,780) containing the 1 kb region that was used for the phylogenetic analysis of the sacsin-like gene in a previous study^13^ was selected for sequence determination. Genomic sequences from the same *Acropora* individuals and from a *M. aequiturberculata, M. efflorescens, M. spumosa,* and a *M. cactus* (see Table S1) were used to design conserved primers (Table S2).

Approximately 1.2 kb was amplified using PrimeSTAR GXL DNA Polymerase (Takara, Shiga, Japan) with primers R10iF and R10i2R. The PCR conditions were as follows: denaturation step for 1 min 30 s at 93 °C, followed by 30 cycles of denaturation for 30 s at 93 °C, annealing for 30 s at 55 °C, and extension for 1 min 30 s at 72 °C.

All PCR products were cloned into pMD20 vector (Takara) using an In-Fusion HD Cloning Kit (Takara), and the sequences were verified using an Applied Biosystems Automated 3130xl Sequencer (Applied Biosystems). The PCR amplification and the PCR product cloning into a vector were performed once for each sample. To remove PCR errors, when the identical nucleotide sequences we determined from more than three independent clones, we used the sequences in our study. The nucleotide sequences were deposited in GenBank under accession numbers LC598237-LC598242 and LC598244-LC598248.

### Determination of sacsin-like gene sequences from short reads of A. millepora

Genomic DNA reads of 12 *A. millepora* were downloaded from SRA and mapped to the genomic assembly version 2.01 of *A. millepora*^13^ with the same mapping conditions mentioned above. Mapped reads to the 1.2 kb locus used for PCR amplification were checked manually to select homozygous individuals (homozygous at the 111 sites that separate two haplogroups) of the haplogroups at this locus, and two consensus sequences were extracted from each of two individuals (HH16 and CS11) using CLC Genomics Workbench with the same conditions described above.

### Examination of inversion on upstream and downstream sequence of the sacsin-like gene

To verify whether there is an inversion including the sacsin-like gene, upstream and downstream sequences of the genes were examined using the following PCR analysis. Genomic DNA of a heterozygous individual of haplogroups 1 and 2 (Adig3) of *A. digitifera* was used as a template for PCR. We hypothesized inversion boundaries on genomic regions where the nucleotide diversity (Pi) becomes low and designed primer sets upstream and downstream of the hypothetical inversion boundaries, respectively. These sets can amplify the target sequence when the template genomic DNA does not contain an inversion of the sacsin-like gene (Fig. S2a–c, primers in upper panels). If the template genomic DNA contains an inversion of the sacsin-like gene, the PCR product will be amplified by the primer set for amplification of the inverted region (Fig. S2a–c, primers in lower panels). Four primer sets are capable of amplifying the target sequence when the template genomic DNA does not contain an inversion of the sacsin-like gene, and six primer sets are capable of amplifying the target sequence when the template genomic DNA does not contain an inversion of the sacsin-like gene were used for PCR analysis (Table S3–4). To check the quality of genomic DNA, a primer set (34639_F1/34639_R1)^19^ was used to amplify elongation factor 1-alpha: LOC107334639 as a positive control. The PCR conditions were as follows: denaturation step for 1 min 30 s at 93 °C, followed by 35 cycles of denaturation for 30 s at 93 °C, annealing for 3 min (PCR#1,2,8 and a positive control) or 8 min (PCR#3–7,9 and 10) at 55 °C, and extension for 10 min at 72 °C. The results of PCR were confirmed by the electrophoresis patterns of PCR products.

### Construction of phylogentic tree

An ortholog of the sacsin-like gene in the genus *Astreopora* was used for the outgroup. *Astreopora* locates as a sister clade of the clade that includes *Acropora* and *Montipora*^14^. An ortholog of the sacsin-like gene was searched by tblastx search^20^ using the CDS of the *A. millepora* sacsin-like gene as a query against all CDSs of *Astreopora myriophthalma* genome (GenBank: BLFK00000000.1). A CDS that was a top hit of the tblastx search^20^ was used as a query of a tblastx search against all CDSs of *A. millepora* genome to test the reciprocal best hit of the sacsin-like genes in *A. myriophthalma and A. millepora*.

Partial sequences of the sacsin-like gene were aligned with the sacsin-like gene sequence of *A. myriophthalma* (astr_s0055.g81.t1) using ClustalW in MEGA ver.7^21^. A phylogenetic tree was constructed using the Maximum Likelihood method based on the Tamura 3-parameter model^22^ with 1000 bootstrap replications.

## Results

First, we verified the positions of the CDS of the *sacsin-like gene* in the *A. millepora* genome using mapping RNA-seq data. Results indicated that the *sacsin-like gene* is composed of three exons on chromosome 7 (Fig. S1a).

In mapping this region, we observed four indels that did not match the mapping results of the short reads (Fig. S2b). We corrected these sites when we translated the CDS to amino acid sequence. The translated amino acid sequence of sacsin-like protein contained a conserved domain that is structurally related (> 90% confident in Phyre2) to the nucleotide-binding domain of Hsp90, a J domain and a HEPN domain (higher eukaryotes and prokaryotes nucleotide-binding domain).

**Figure 1:**
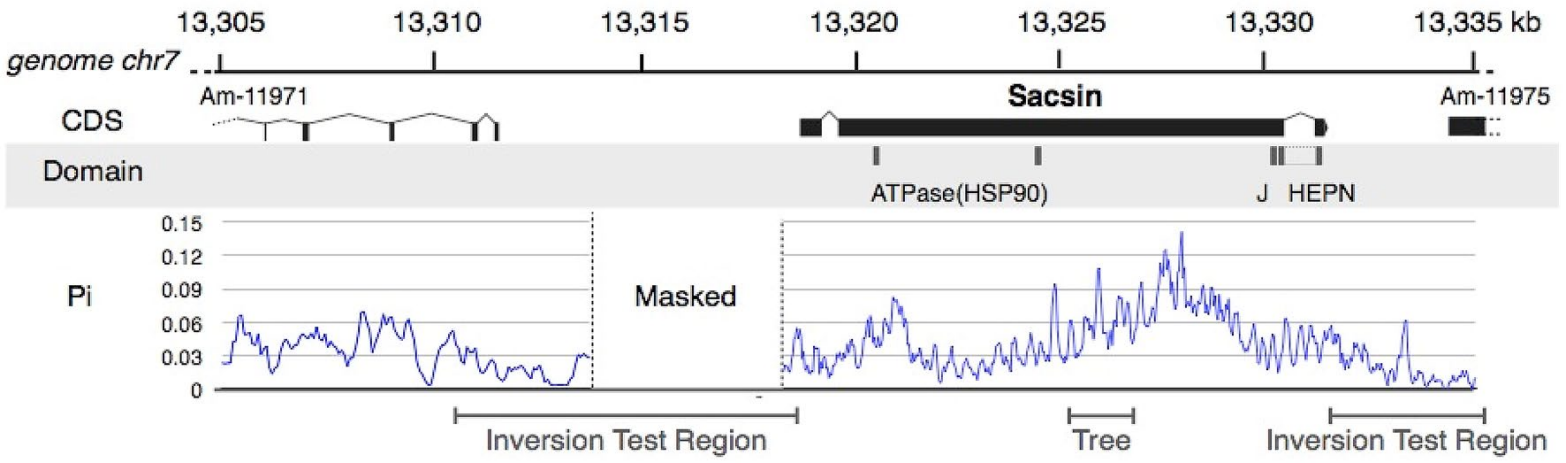
Gene structure and nucleotide diversity (Pi) of the sacsin-like gene. The predicted CDS structure of the sacsin-like gene on chromosome 7 is shown, as well as the flanking downstream gene region. The positions of conserved domains in the sacsin-like gene are shown under the CDS structure. Values of nucleotide diversity estimated in sliding windows of 100 bp with a step-size of 25 bp are shown. The bar labeled “Inversion Test Region” indicates the region where the PCR was performed for the inversion test. The bar “Tree” indicates the region used for the construction of the phylogenetic tree.

In a previous study, the elevation of nucleotide diversity (Pi) in the sacsin-like gene compared with surrounding regions among *A. millepora* was reported^13^. To test whether such elevation of nucleotide diversity (Pi) is also observed among other *Acropora* species, we calculated Pi among *A. digitifera* in a region containing the sacsin-like gene. As in *A. millepora, A.digitifera* showed an increase in Pi between the Hsp90 and J domains in the sacsin-like gene (chr. 7; 13,325–13,330 Kb) compared to the surrounding region (Fig. 1), indicating diverged haplogroups of this region exist in *A.digitifera*.

We cloned and sequenced approximately 1.2 kb partial sequences of the sacsin-like gene from *A. digitifera* (n = 3), *A. tenuis* (n = 2), *M. aequituberculata* (n = 1), and *M. informis* (n = 1). One sequence was determined from a single individual of *A. digitifera, A. tenuis* and *M. aequituberculata*, and two different sequences were determined from two *A. digitifera* individuals, a single individual of *A. tenuis* and *M. informis,* respectively (Table S5). These results suggest that this sacsin-like gene is a single copy gene in these four species as with *A. millepora*^13^.

For *A. millepora,* we extracted a consensus sequence of the sacsin-like gene using NGS data of *A. millepora*, because we do not have genomic DNA of *A. millepora*. From the 12 samples (Table S1) used in the previous study^13^, three (HH16, TR02, and FR20) were predicted to have one homozygous haplotype of the sacsin-like gene, and one sample (CS11) was predicted to have the other homozygous haplotype. Consensus sequences were extracted from two individuals (HH16 and CS11) with different haplotypes.

**Figure 2:**
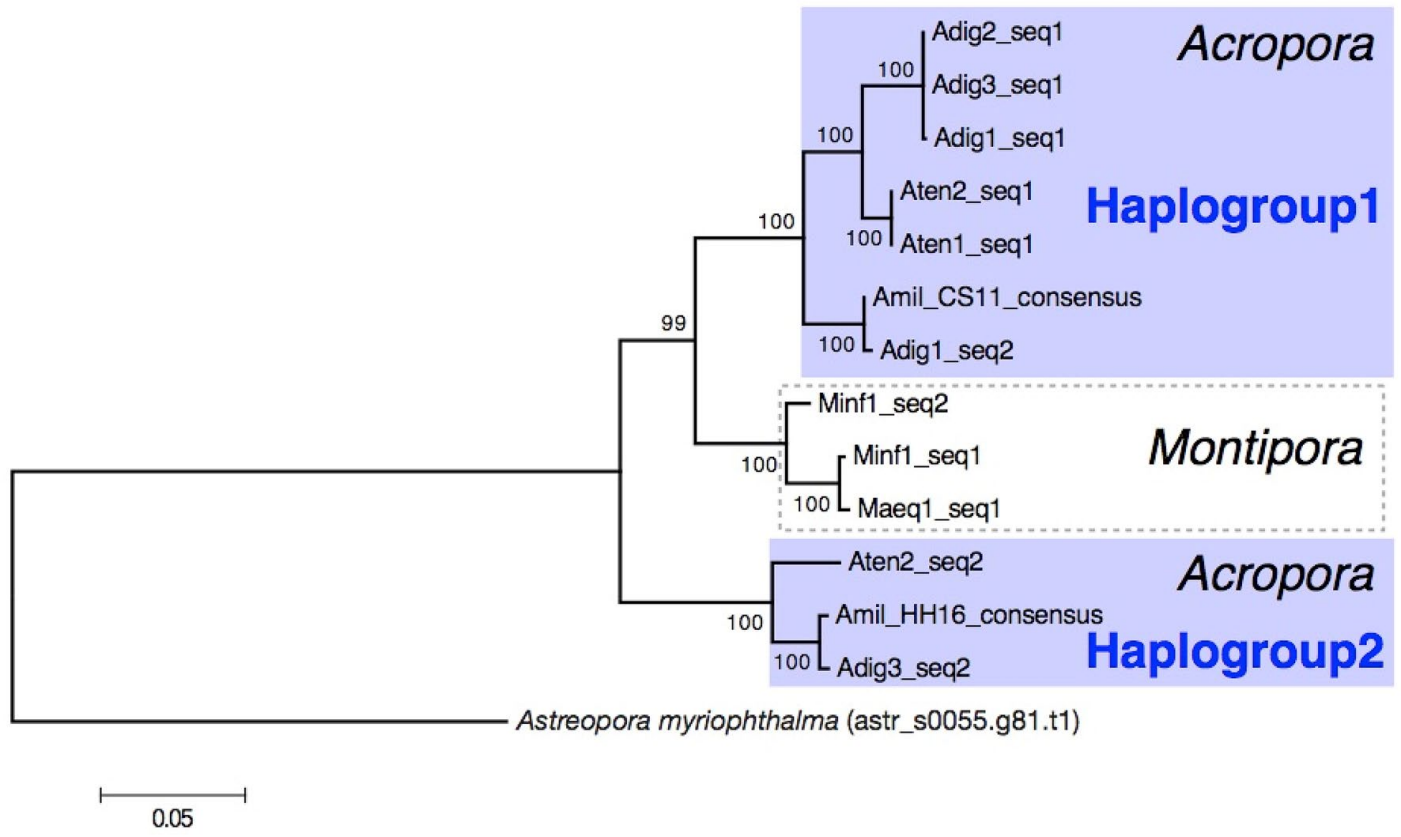
Phylogenetic tree of partial sequences of the sacsin-like gene.

We constructed a phylogenetic tree using 11 cloned sequences, two consensus sequences, and one sequence of the sacsin-like gene from the *A. myriophthalma* genome assembly as an outgroup. Sequences of the sacsin-like gene from *Acropora* species formed two clades (Fig. 2) and we refer to these two clades as Haplogroup 1 and 2.

All sacsin-like gene sequences isolated from *Montipora* formed one clade and this clade clustered with Haplogroup 1 (Fig. 2). This suggests that two haplogroups split before the divergence of *Acropora* and *Montipora* (119 Ma^14^). No sequence from Haplogroup 2 was found in *Montipora* species.

Phylogenetic gene tree based on 1251 bp of a sacsin-like gene region. The tree was constructed using the Maximum Likelihood method based on the Tamura 3-parameter model. Bootstrap probabilities obtained by 1000 replicates shown next to each node. The scale bar represents 0.05 substitutions per site.

Chromosomal inversions are a common mechanism to avoid recombination and to maintain diverged alleles^23,24^. We tested for inversions within the sacsin-like genes using PCR. All primer sets for amplification of the sacsin locus without inversions amplified PCR products (Table S3, Fig. S3). In contrast, the primer sets for amplification of the locus with inversions either did not amplify PCR products or amplified PCR products of many different lengths, inferred to be non-specific amplification (Table S4, Fig. S3). These results suggest that, at least in the genomic regions tested in this study, two sacsin haplogroups do not have chromosomal inversions.

## Discussion

*Acropora* corals produce dispersing larvae that may experience strong selection in each generation from stressors operating in their settled environments^13^. Alleles related to stress response in different environment may have been maintained over long periods by balancing selection. In this study, we showed that two divergent sacsin-like haplogroups persist in at least three *Acropora* species. Although we analyzed two *Montipora* species, no sequence from Haplogroup 2 was found in *Montipora* species. This may be due to two possibilities: either the individuals of *Montipora* species used for PCR analysis were homozygous for Haplogroup 1, or Haplogroup 2 is lost in *Montipora* species.

High temperature experiments with *P. acuta* suggested that sacsin may be involved in the high temperature stress response^10^. Sacsin is a member of the HSP40s family, and some Hsp40s may recruit an Hsp70 to a specific set of substrates^9^. The sacsin-like gene of *A. millepora* contained the nucleotide-binding domain of Hsp90, a J domain and a HEPN domain (higher eukaryotes and prokaryotes nucleotide-binding domain).This is consistent with the structure of human sacsin, except that human sacsin proteins have a ubiquitin domain^25^. Two divergent sequences of the sacsin-like gene may be involved in the recruitment of Hsp70 to different substrates under stressful conditions such as changes in sea water temperature. The ancestors of corals have experienced periods of high seawater temperatures^26,27^ during which two divergent haplogroups of a sacsin-like gene have persisted with some potentially adaptive functional role in different habitats (possibly different water temperatures) to play.

Chromosomal inversion can help maintain two divergent haplogroups. For example, alleles of a 4.5-Mb region which control different morphs in the ruff (*Philomachus pugnax)* have been maintained for more than 3.8 Myr and these alleles are located on an inverted region^23^. However, at least in the genomic regions tested in this study, two sacsin haplogroups do not have such inversion. As reported in *A. millepora*^13^, the sacsin-like gene may have been maintained by selection to respond to different environmental stressors. Although the genetic mechanism by which diverged haplogroups of sacsin-like genes is maintained remains unknown, the long-term persistence of haplogroups suggests that sacsin-like gene diversity may function in local adaptation to heterogeneous environments with different heat stress factors. The divergent haplotypes of the sacsin-like gene in *Acropora* corals will facilitate our understanding of the stress response of corals to environmental change.

## Supplementary Information

Supplementary Information.

## Data Availability

DNA sequences: Genbank accessions LC598237-LC598242 and LC598244-LC598248. A modified CDS and consensus sequences of the sacsin-like gene constructed from *A.millepora* are shown in Additional File1.
