# Supplementary material for: Two divergent haplogroups of a sacsin-like gene in Acropora corals
Source: Sci Rep. 2021 Nov 26;11:23018. doi: 10.1038/s41598-021-02386-w (PMC8626496; doi:10.1038/s41598-021-02386-w)
Supplement: Supplementary file 1 — Supplementary Information. [file 41598_2021_2386_MOESM1_ESM.pdf]

Supplementary Material for

Two divergent haplogroups of a sacsin-like gene in Acropora corals

Shiho Takahashi-Kariyazono[a*] and Yohey Terai[a*]

[a]SOKENDAI (The Graduate University for Advanced Studies), Department of Evolutionary Studies of Biosystems, Shonan Village, Hayama, *240-0193,* Japan.

*Corresponding author: Y. Terai and S. Takahashi-Kariyazono, SOKENDAI (The Graduate University for Advanced Studies), Department of Evolutionary Studies of Biosystems, Shonan Village, Hayama, Japan, +81-46-858-1572, terai_yohei@soken.ac.jp, takahashi_shiho@soken.ac.jp

This file includes:

Sequence data

Description of the script

Figs.S1 to S3

Tables S1 to S4

1 Sequence data

Three sequences: CDS of the sacsin-like gene in the *A.millepora* genome (chr7:13318370-13318907, chr7:13319250-13330216, chr7:13330968-13331252) and two consensus sequences extracted from each of two *A.millepora* individuals (HH16 and CS11) are shown below.

```
>Amil_sacsin-like_CDS
ATGAGACTGAAAGAACCTTTGTTCAAGTCACTGTGCAATAAGCAAGTCATCCATACCGAGGCATGTGGAGGGAAATGG
CTCTACGTCGGAGAAGCTATCTTTGACCGATTAGCAGAAAACGACCCGGTTGAGTTGCTTCGAAGCCTGCTTCTCAAA
GCCCATGAAAACATCGCCACAGTCCCCAGCCACGTTTTGCAATCGATTCAAGACTTTGAACAATTTATCACAGAAGTG
ACACCTTCACTTGTACGTTCGGTTCTCAAAACAGTGCCATTTTCGTatgaaagtcttgaaaaagaagagaagctgCAG
CTTCTCAAGTTTGTTCTCAAAGACGAATGCTTTTCTGAGCTCTCAGGCCTAAAACTGTTGCCCGTGTCTGATGGAGAA
TTTGTACTCTTCTCAAATTCAGATGAAACCATTTTTATTTCATCACGGGAGCATCCCCCCGGACTAATACCGACATTG
CGTCACCGCCTTCTGGACCAATCCTTGGAAAGTGAAACCTTGACAAAACTGGAAGCTGTTGCAAAAGCAGAATGCACT
CAGTTGAAACTTCTTGAGAGATTCCACATACCCTCTCTATTGCGAGAATCGTTATCTGGGAAATTGACAAATGAGGAT
CTTCTCGACGGGTATCCGGTAGACCAGTGGCTCAAGTGCCTATGGGAGTACTTAGGACAACATTTTAAGACGCATGAA
GACCTCTCTCTGGTGAACAACCTACCTTTAGTACCAGTTGATCCATCGAAAGGCGCCCTGACAATGCTAGCAAACCCG
TCTAAAGTTGTTGTTAGATGTCTGGATGACCAACGCTTAGAGAAGAACGTGAGCTCAGTGTTAGAAAATTTCGGGGTG
ATTGTCTTGGAATCTTTGCCAGACTACTTGAAACACCATCCCTGTGTTCTGGATACATACGTGCATCGACCGTCAGTT
CACGGTGTTTTGCAAGCGATGGCAGTTTCAGCTTCTGATTGTCTAGGAATGCTTTCAGCAGTCTTGCTGGACATGCAA
GAAAAAGGTAGAGGTGACGACTTACTTTCACTGAGGAAATTTATTTCTAAGACTGAGTCACTGGAGCCcagagaaaaa
gaaattattagcTCGCTCCCACTTTTTGAGGAAACTGGACAGCCACATTCCTTTGTATCAAAGAAAGACGTTTGGGGT
GCTGCACCACAAGATGCAGACGACTATTTGGTGGCTCTCCCCACTGCAACCAAATTTATTGACACAAGAGCTGACGAC
GCAAGACGGTTAGTTACTTTGTTGGACATGAAACCCGTAACAATTATTGATTTTCTTCTCCACGGGATATTTCCTTGT
GTTCGCGAAGACGCATATTGCAATGAAGACATTGACAGAGTTATGAATGTTGTTATCAAGAGATACGATATTCATAGC
GGTAACCGAGCGAGATTAGAAGAAGAGATGAGAGATCTGGCCTTTGTCCCGACCAAACATCGTCGTGTTAAAGCAAGG
GAAATCTTTGATCCAAGGAATGAACGTCTTTGTCACATTTTCGCAGAAGAAGACGTCTTTCCAATTGGAGAGCAATAC
AATGATCCAACAGTCCTCGCAGTTCTGCAGAAACTTGGAAtgaagagcgaaaatgaaataACTGCTCAAGATCTCTTC
CAAAGTACCCGCACAGTTTCCGAAATTATCAACAAGAGAAGAGCAGAAGTCAAGTCGGAAGCAATAATGGTATACCTG
GAGAGTTATCCTGACAAGCTTGAAGAGCCAATAAATGGAAAAGAATTAGGGGATATACTTCACGAGACTTCTTGGATA
TCCAGAATTAATCAGACGCCTGACAGGTTTCCCAAGAGCCTCCCTTTCATTGGCGCGACCGAAGTAAAGCCCAGGTTT
```

TACAAGCCTGCAGAAATTCACAGTGCAGATTTTGTTGACATTATTGGATCCGTGAGACCGATTGTCAAAGTTCAGTCT

TGTGGGAAAGTAGCCAAGCATTTTGGCTGGGataaaaagccacaagtcaccGTAGTTGTGAAGCATTTGAAACTCGTA

ATTGACTCTTACAGTCAACAAGAAAAGTCGCTTTACATGATGATGGTGGCAAAGCTATACTCTTTCCTCGTTGATGCC

GAATACGCTCTTGTCAGGAACTCCTTTGAAGAAATGAACATTGTAAGATGGATCTGGAATGGAGATGGCTTTTCGGCT

CCCTCAGAAATACTTGCTGAAAAATCACTCTTTGATTTATCTCCTTACATCCTGTCCCTTCCTCCAGAAATGAAACAA

TATCAGACATTCTTTGCAATGCATGGCCTAGCGGTTGAATGCGACGCCCACGTATTGTTTCGTGTTCTTCGATTGATG

AAAGAgaaatatgagcaaaataaCCCTCCTGTTGAGGTCAATGATGTGAAGCGAGATTTGCAGCTGTCTATTAACATT

TTAAATGACTTAAAGAGTCGTAACATTGAACTCCCTTCTTTGGGCGAAGAAAATGTGCTTATTCCAACGTTTGTAGAA

GGAGATGAGTTTGTGAGGCTTGCACCAGCTGAAAGCTGTGTCTATTGTGAACGCGAGTGGCTGCAACAGGAAAATGAT

GAAGAGGAAGATGGTTATTTCTTCGTCCATCCGTCGGTTTCTAATAGCACAGCAGAGTTCTTCGGTATTCGAACATTG

GGGCACGTCATGCTCGATCCCGACGAGCTAGGAGTAGGCGAAGAATTTGGACAGGAAGAAAAACTTACCCGTAGACTA

AATAGGCTTCTGGAAGAATACACTGATGGGTTCGCCGTGCCAAAGGAGCTTATTCAAAATGCAGACGATGCAGGTGCA

ACGGAAATCAAGTTTCTTTATGATGAGCGTCAAAATGAGGATGCCCTAACTTGTCTCATTGACGACGGCATGAGAGAG

TGTCAAGGTGCTGCACTGTGGGTGTACAATGATGCGGAATTCCGCGATGAGGATTTTGAAAACTTGACAAAGTTAAGT

GGAGCGACGAAGGAACATAGCACAgagaaaattggaaaatttggCCTTGGCTTTAATGCAGTGTACAATCTCACAGAT

GTCCCCATGTTGGTTAGCAGAAATTACTTCGTTATCCTTGATCCCCACACCTTCTATTTGGGAAAGGCAATTagaaac

aaaagcaaaccaGGCATGAAAATCGACCTGAATAAAAACGTGAAGAGACTGCGAACATTTCGCAATCAATTCAAACCG

TTTAATGGAATATTTGGTTGCGATCTTGAACTCAAGGAAGAAAGAAACTCGTATTCTGGTACATTGTTTCGTTTCCCC

CTTCGAACCAAGGAACAAGCTGTAAAAAGCGAAATCAAGCAACTTCACTATGACAGCAGCCAAGTGAAAGCACTATTG

CTGAAGTTCATTCTTGGAGCAAGATCACTGCTTCTCTTCACACAAAATATCCGTAAAGTTAGCATTCTTCATTTGCCA

CATAAAGGAAACCAACCGGATGTGATTTTTGAACTTACTAAGGAACTTTCAGAACATGGAATCTTGAAAGAGCTCTCT

GTGCCATTCAGATTGTCCCCTCCCGCTGAGAATTTAAGCGAAAATGATCAGCTCCTTCTAACACAGTGCAACTTTCTA

AAAGCTTCTTCGGAGTTTGTCAAGAGTACGGAGCTTTGCGAGAATTCACGATCTGATTTGCTACAATCAGCGTTTAAA

GTTAATGCAGTTAGCACCGTTTCGAATTACGGACGTCGTTTCTTTGGGGACAAAGAAAACTTACCTTCCGGTGCAGAA

AAATGGCTAGTCGTCTCATCCATGGGCACTGGTGAAGCAATGACATTTGCACAGGCACAGCAAAACAGAGGCCTTCTC

TCAGCTGCTGGAGTTGCTGTTCAATTGACAATTGAACCAACTTCAGTCTCAGCTCCCATTTGCCCTCCCAAATATCCA

GGAGCTGTATTTTGTTACTTACCGCTTCCAATCGAGAGTGGACTACCCGTTCACGTCAATGGCGCGTTTGCAGTAGCC

TCCAACAGACGTTCTCTAAAAGAGAAGACGGAAGATGACAAAGATTGCACTGGAGTAGAGTGGAACGACGTTCTCTTG

AAAGATTCGGTTTGTGCTGCGTATCTTGACCTCATCACACACCTTAAACCAGCCATAAACAAGCCTGGAAGTGGGTAT

CTGTTCCACTCTTTGTGGCCAAGGGACTGCAAAGTGCAAACAGCTTTGACGCCTCTTTCACGTTCTTTTTACGAAAAT

CTTGTTCACAcgaactttccatttttttcaaatGGTACCAGAAGGGTTGGAATCAAAAACGTTGTCTTCTTTCATCCA

GAGTTTCGAAATGATGAACAAATAGGAAATACAGCATTTGAAGTGCTGAAGTTGTTAGTACGTGACGATAAAGCTGTT

ATTGATCTTCCGCTTGAAGTGCTTGACTCGTTTAAAAACTATGGTCTTTTTCAAGAAATCCAATCTGGACAATTTGAT

GGGATGAGGTTTTTCCGCGAATTGTTTTTCCCAAACATCTCCACTCTGCCTCAGCAGTTAAGAGACAACTTAGTCCTA

TACGCTCTGGatgacaaacaaaagaaatttgatgatTTGATGAAAGAGTATGAATGCATCCCTACATCCCCTAGGGGA

ATAAGACTAAGACGTCCTCAGCATCTGGTCAGTCCGAAACGAGAAGTGGGTCTGCTGTTTTTGCCACAAGATGAAAGG

TTCCCGCATGGCACCCAAGAGACATTTCTGGGTCCTATAAGGATGTCGAATCTTGAGCGACTTGGAATGTTGACTGAT

GATATTCGTTGGCTGGAAATTGCAGAAAGGTCGGAGAGTATTTGGTCCTTAAATCAAGAAGATAGCACTGAAGCGAAA

AAGAGGTCAAAAACCTTGATCGAATTTTTGGACAAAAAGCTGAAGTGTGAAGAACCTCCACCACAAGAAATTAAAGAC

AGGATTCTCCGGGCAAAATTCCTTCCAGCTCTACAGAAACCACCGTCCTTCCCCCTCTCTTGGAAGGGAAATCAAATA

CGAGATGGAAACAGGCAAGTCCTTGTGGCGCCATCGGAAAGCTTTttgaagaaggaaaagtatttaGTATGTTGCAGT

GAGGTCCTAATAGACCTTCACATACCAGAGGTTGTGCAAGAATTGTTGATGTTGAACAGCAAGAAGCCAACTGTAAAG

CATGTAATGACTCAACTTAACGAGGCAATTGCTGCTGTCTCAAGTAGCTCTGGATTTGAAGAATTGAGGAAGACTTGC

TTGAAATCTTACAAATATCTTCAAGAGGCTCTTGAGGATAACCAAGGAGAAATTTTGCATTTACTTAAAGAGAAAGAG

TTTATTTTGGTGGGAAGGAATTTCTGTAGTGCAAAACAAATTGCGTTCAAGTTGGACACAGATTGCTCACCTTATTTG

CAAAAGGTTCCTGATGAACTGGTCAGAACGTGTGGGAACTTTTTGAAAGCAGTTGGCGTAAAAGATGTTTTCTGTGCG

AAAGATTTCATTGATTCGTTGGAGTGCATTAAACACAAATTTGGAGAACAAGCGCTAGACAGGCAGTCGCTTCATGTC

GCTGTTCTTTTGGCAACACAATTAGAAAAATCCCTTGAGGATCATGAAGTTACCATTCCAACTGATAAGAGGAGAGAA

TCTATCTATCTCCCCAACTCTGATGGAGTAATGCAGCTGGTGAAGGATCTTTGTTTCAACGACTGCCAGTGGATCTCT

AAAGTTAATAATGTCAAGTTAGTCAGTCCAAAAATCCCACCCTCAACAGCTACCATGCTTGGAGTCAAAACTAAAAGG

CAAGAGGCTTTGCGAAAACGCACTCTCGGAATATCGTTTGGTCAAAGAGAGAAACTTACAAACCGCTTGAAACGTATT

CTCACTGGCTATCCCTGTGGTAAGGAACTATTAAAAGAACTAGTACAGAATGCAGATGACGCAATGGCAACAGAGGTC

TGTTTCGTTAAGGATCCTCGCACCCACCCaaagaaatatatttttgaCCCTTGCTGGGAGCCACTACAGGGTCCAGCA

TTGTGCGTGTATAACAACAAACCTTTCAAGAAGGTCGACATTGAGGGTATACAAAGTCTTGGACAGGGCAGTAAAGGT

GACGATCCAAACAAAACAGGCCAGTATGGCGTGGGCTTTAATGCTGTCTATCATTTGACAGATGTGCCTTCTTTCATG

TCTCAAGGAGAAGAGATTGGTGAAGTTTTGTGTGTGTTTGATCCACATTGTCGGTATGTACCTGACGCCAACCCTCAA

GAACCAGGAAGAATGTACAATACCGAGGACATGAAGGAAATGTTTTCAGATGTGTTTTCATGTTACCTAGGAGACTAT

TTTCCCCTCAGGAATTCAACAATGTTTAGATTTCCTCTTAGAACGCAAGCAATGGCAGACAACTCTCAGCTATCAGAA

ACGGAATTTACACTGAATGATCTGAGTAAATTAATGAAAGCACTGCAGAGGGAGCTTTTTGAGGTGCTTCTTTTCCTA

CACAGCGTGAAAAAGATCACGTTGTGCGACTTAGATGAGAGTGGAAATGTGTTGAATTCATACTCTGTGGAAGCAGTT

ATATCCCATGAGGATTCAGTAAAACAGGAGGAATTTAACAGGCACAGAAAGGAGATGGGAGAGGAGATGAAAGTAAGG

GGATTTCCTTCCAAAGCAGAGACGTATTCGTGCTCTTACGTAGTAACCTTAAACGACAGTTCTGGAAACAGGGAGGCG

TGGTTCATTGTTCAGCAGCTGGGGTTCGAAAACGATGTTCCAAAAAGTGTTGTTGAAGCCTACAGGAGACACGATCTT

GGGATGCTTCCTCTGGGAGGAGTTGCTTGTCTCTTGGAGCAGCAATCAAAAGGGAGAAAAGGTATGTTGGCTCAAGGA

AGGGCAAAAGCATTTTGCTTTCTGCCACTTCCCTTAGAAACCGGTCTTCCAGTGCACGTAAATGGTCACTTCGCACTG

GATCATGAAGCAAGACGACACATGTGGTGGGATGAAACTGGCGGATACCGAACTGAATGGAATGAAGTTTTGTTAAAA

GACGTAATAGCATCTTGCTATTTAAAGCTTTTGGCAGAGGTACGTAGCCTCTACCACCCCAAACTCATCCAAGGAGCA

GAGCCTCTGAACAGCCCCGAAGAAGTCCTAGTGAAAACACTTAAGGACTACGAGAATTTGTTTCCAAAAGTCAGTCCA

GCGAAAGGTGATTATTGGGTGTTGCTTGTAAAATCCTTGTATCggaaaatgaatgaaaagcAGATGCGACTTCTTCCA

GTGGTGAGGGAAGTACCAAAGAAGCAGCAAGTGCAAATAACGTGGCTCCCACCAAGAGGATCTGGAAAGGACCAAGCT

TTCTTCAATAATCTCAAATCTGATGAGAGACCCAAAGGAGAAAAGCAAGAAGAGATGTCTTTGTCAGAGATTTTGATT

CAAATGGGTTTTAACCTCGTGCGTTTTTCGTTGTCTATTTGGGATGCGTTGAACAAATCTGGTGTAACTGCCACCTGC

ATCTCGCCGTCATCAGTAATGACCTTTTTGAAGGGCTATAGTTCTCAAGATAGTCATTGTAGTATTGGATTCATACCA

ACTGATGTCAGCAATACGCCTTTGTTGACTGAAAGAGGCGTGGAAGAGGTGCTAAGATACTGCAAGGATGCTGAAGGC

TTTTACGATGACTTATCTGGTCTCCCCTTACTTCTCACCCAAGACAACGTCCTTCGTGTGTTTGACGCAAGAAATCCA

ACATTTTTATCGCGTTACCATGATATTTTGCCACAGAGTAAAGAGATGTTTGTTCATGATCGACTGCGAAAGAATTTA

TTTCACGATCATAGAAGTCAGAATGCCCATGTGTTTCAACCCTTTGACGTAAAATGTTTTGCCACATATTTGCATCAC

ACACTGCCACCAGCATATCGCGGAAGAACTAATTATGTGAGCTGGTCTCCAGATCAGCGTTCCGATGCTGAGCCAAGT

CGTCGTTGGATTTCCAGAGTATGGAGCTTTCTACATGAAATCGTGAAGGCTAAGGAGTTAGAGATGGGAGGTTCAAGA

TGCATTCAGGAAGAGCTAGAACCCTTGCTCAGTTGGAGTATTCTTCCAGCGAAAACGCAAAGTACAGCGTTGCAAGGT

AGTAGCAGCTTAAACTTTACTATCCCGCAAACAGCTAGAGTTCTGGTTCCCCTGAAATTGGCTAAGTCAATTTTAGAT

TGCGCTTACTGGGAAATGGTCCCATTGCATAAGCCTCTGGTCCACGTCCTTCGAAAACTGAATCCCCCCGAGCTTGAT

ACTTCTGTTTTCGGTGACAGTTCGTACACGCAGCTTGCGTCTCTGGCACGAAATTTCATCGCCTCGTTAGAAATTCCG

GAATCCCTCCTTTACGCACTTAATCATTGGATGACAACTCATCCCAACTGTCTGAGGGATAAATTGAGTCCTGACGAC

TGCAAGACATTGTTGAAATATTTCAGCGACAATGTAAACACCCTGAAGTCTACCAACGCTGAGAGGGAAATCTTAAGA

AGGCTCCCATTTTATCTTTCGACCCACGGCAATCTCATCAGTCTTGACAACAAGCCAATTCGCCTTCTACCAAGTGGA

ATACCAAGAGTGGGAATAAGAACTTTACAAGGACAAGTCGATGTTCTTTTTCTAGAATCTGTGGAAGGTCTCTCCAGC

CTCTATactttttgaattttgatacAGTCTCTCAAGTCAGCGTTTACTGTGAGTTTATCTTCCCACATTTCAAGCTC

CTCAGCAAGGAAGCAAGGGAAATGCACCTGAAGTATGTTGGAGAAGTTGTTTTAGCAAGTATTTTCACGACTGATGAT

gacaaattgaaaataataaactgTCTAAGGATATCGGAAGTTGTCACCGTGGAGGATGGTTCCTTGAAAAGAGCCAGT

TGTTTTTACGACCCTTTTAACGATGTCTTTAGGATCATGCTTCCAGAAGATAAATTTCCACCGTACCCTTACAGGTCA

TCGTTGTGGTTGCCTTTTATGAAGAAGATTGGAATGGTGCATGAAGTGTCTCCCACCTTATTCAAAAAATTTGCCAAG

GACATTGCTAGAGAAGCCTCTGTGCAACCTACCGAGAAAACAGACCGAAAGTCTAAAGTGCTTGTTTCATGTCTCTTC

AAGCCACCTCATGTGAATGATGCAAGTCTTCTGCAAGATATTAGTGGAATTCGCTTCGTGATGTCAGATTCTGTTCGT

TCTGACCTGCTACAATTACATTCACAGTATGAAGATCAGCATAGCCATTACATTTCATTCAATGACTCTGTTGTTGAT

GAATTTACCGAGACTGTGTGGACAGTAGCACATCTTCTCCCGCGCTGGGCAGACCCGAGACCTCACAGTTTCCAAAAC

AACTTTCTCCTTTCGAGACTTCATGTTTTGTCACAGCCCAGCCCAGAAATGGTTATTTCTCACTGCTCTGTTTTATGT

TGTCATTTGGCAAAACAAAACGGTGACATGATTTCCGACAGTGAAATTGCCACCAGAAAGTCAGTCATGCGAGAGGTG

TACAAATTTTTCAGTGACAAAGTTAGTTCAAGTTATAACCTGAAGGAGAAGCTTGTAAACGTACCTTGTATTTTGGTT

GAAGATGGAAGGAGGTTTGTGCTGGCTAAGCAAACTGTGCTTAACCTTCTCGAGGAGGATGAGATACCTCCCTTTCTT
TTCCGAGTGCCTTTCGAGTTTGGGGCGTATCACTTATGTTTTCAGAGTTTAGGGTGTTCAAAATCCGTGAAAACGTCT
CACTACGCGTTCCTTTTGGAAATGGTGTACAAGCAGTGCAATGGAAAAAAGCTCCTCCCCAAGGAAGTTGAAAGCTCC
TTAAAGGCAGTAAGAGGTCTCTTCGAAAGGCTTGAACATGATTCAGAGGAAGGCGTTCATCTCTCAGATGTGTACTTG
CCAGCAGTTCATCGATTCAACAGCGATTCGATTGGGAATCCGTCTGTAACCTTGCACAAGTCCACTGATCTAATTTTC
GATGACGCACCCCGGTATCGAAGCCGACTTGGAAACTTCAATGAATTGTTTGTGGTGGACCTCAAGAGGACCGGATTG
CAGAGCACGACATTGACGAACTACAGAGATTACATCATTCGCCTTCCAAGCGAATCTCGAACAAAGATGCTATCAGAG
GAAGTGAAGGAAACGCTTGTCCACCCTACTGAACCTGTAAATGATAATGGCATAGCTCAGTCGTTAAAAGAACAACTT
TGTTCAGAGCAGTTTATTTATGGCATTGTAAGACTACTGCGCCACGCAAACCCGGACAAACCACATCTAGTCGAAACT
GTCAGTACGACATTAGAAGCAAAACTGAGAAGCATCCAGATAATCGGAAGACCGAAAATTGAAACCCACCTAATCCAC
GAAGGCCATCTTATACCAGGAAGCCAGACGGAAGTGGCATACTTTGTTGACAAGACATTAGATGGTGACGAAGCCGTT
TGGAAGCTCTACGTTAATGCAGAAGCAGAGGAAGATCGTGGTAAAATATGTTTGGTCTTAACCCAAGTTATAGCCGAG
GCATGTGAAGGGCTGCTACGCGATGCAGTGGCTTATGTCACAGAGATGTTACGCACAGACCCTGCCAAGATATGGTCA
GTATTAGACGACATGGAGATTCGCCAAGACGACTCTTACGATCCTTTAGATGGCAATGTGTTGCCTCAACCAGGAAAT
TTCATTCCGATTGAAGACCATCACCTATTGAACGAAGCATTTGAAGAATTTTCACAAGGGGAATATGTTGGCTTAGAA
TTGGAGGATCCTAGTCTCGAGCAGAAGGATGGAGATGCTACTTTCATTTACGCTATCATAACTGGGGAAGTAGAAGGC
CAAAAGGATGCTACCCTGTACACAAAACTTTACCTGGTTAAAGTAGATCGCGACAGAGAACCCCAGCAAAAGGAATCG
GCTGATTTGTACAAATTTCATCGTATTCAATCAATTGATGAAGCCCACCAAGAGCGAACCGATTTCTCAGTTGAACAG
AAAACTACGATTTTGGCCCATATCACGGAGATGATGGAGAATACCTTTCGTTTGCCCGAGCATAGACGGAGGAAAATC
ATCAAGCGATTATGCTTACAATGGCATCCTGAGAAGAGTCGCAAAGATGAAGCGTTTTGCCATCAGGTTTTGCAGCAT
TTAAAGAAAGAGGTGGCAAGATTGAAGCAGGTTGAGCTACAACGAACAGGCTCCGAAGCGGAAAGCCGCCAAGAAAAG
CCATACGAGGCTTTCTTTGACTTGTGGAGAAAACGTGCCCAGAGACATCATGCTTCACGACAGTCTTACAGAGAACGT
TATGAAGCTGCTGGTGGTGCATCAATGCTCCAGAGGAATAAGTCTGGAATTCCGCCGAGTTTTTCTAAGAAGAACCCT
CAGCCGGGTGAGGCAAGGCGCTGGTTGAGACAGGCCGAAGCCGATTTAAGAGCTGCCGGGAAGGATTGGATGACTAGG
AATCCGTCCTACGAGTGGGCGTGCTTCAAGAGCCACCAGGCAGCAGAAAAGGCGCTCAAAGCTGCTCAATATGCAGAT
GATGCTTTTAAGACTCATGCTCATAGTCTTAAGAGGATTGTTTCTGGATTGGGTGACTCAGAATTACTCGAATTAGCA
AGGCAACTGGAGAACCTTGTGGTGGATTCTACGCGCATGCGCTACCCGGACCAATTATGTTATCCTCAGATTCCTAAC
GACGTATACAGTGGCGAGACGGCCGCGACGGCTCATGAATTAGCCAGAAAGATTGTGGATAAAGTCAGGAAAAAGGTT
GCAAAGTGGTAG

>Amil_HH16_consensus

ATGAGACTGAAAGAACCTTTGTTCAAGTCACTGTGCAATAAGCAAGTCATCCATACCGAGGCATGTGGAGGGAAATGG
CTCTACGTCGGAGAAGCTATCTTTGACCGATTAGCAGAAAACGACCCGGTTGAGTTGCTTCGAAGCCTGCTTCTCAAA
GCCCATGAAAACATCGCCACAGTCCCCAGCCACGTTTTGCAATCGATTCAAGACTTTGAACAATTTATCACAGAAGTG
ACACCTTCACTTGTACGTTCGGTTCTCAAAACAGTGCCATTTTCGTATGAAAGTCTTGAAAAAGAAGAGAAGCTGCAG
CTTCTCAAGTTTGTTCTCAAAGACGAATGCTTTTCTGAGCTCTCAGGCCTAAAACTGTTGCCCGTGTCTGATGGAGAA
TTTGTACTCTTCTCAAATTCAGATGAAACCATTTTTATTTCATCACGGGAGCATCCCCCCGGACTAATACCGACATTG
CGTCACCGCCTTCTGGACCAATCCTTGGAAAGTGAAACCTTGACAAAACTGGAAGCTGTTGCAAAAGCAGAATGCACT
CAGTTGAAACTTCTTGAGAGATTCCACATACCCTCTCTATTGCGAGAATCGTTATCTGGGAAATTGACAAATGAGGAT
CTTCTCGACGGGTATCCGGTAGACCAGTGGCTCARGTGCCTATGGGAGTACTTAGGACAACATTTTAAGACGCATGAA
GACCTCTCTCTGGTGAACAACCTACCTTTAGTACCAGTTGATCCATCGAAAGGCGCCCTGACAATGCTAGCAAACCCG
TCTAAAGTTGTTGTTAGATGTCTGGATGACCAACGCTTAGAGAAGAACGTGAGCTCAGTGTTAGAAAATTTCGGGGTG
ATTGTCTTGGAATCTTTGCCAGACTACTTGAAACACCATCCCTGTGTTCTGGATACATACGTGCATCGACCGTCAGTT
CACGGTGTTTTGCAAGCGATGGCAGTTTCAGCTTCTGATTGTCTAGGAATGCTTTCAGCAGTCTTGCTGGACATGCAA
GAAAAAGGTAGAGGTGACGACTTACTTTCACTGAGGAAATTTATTTCTAAGACTGAGTCACTGGAGCCCAGAGAAAAA
GAAATTATTAGCTCGCTCCCACTTTTTGAGGAAACTGGACAGCCACATTCCTTTGTATCAAAGAAAGACGTTTGGGGT
GCTGCACCACAAGATGCAGACGACTATTTGGTGGCTCTCCCCACTGCAACCAAATTTATTGACACAAGAGCTGACGAC
GCAAGACGGTTAGTTACTTTGTTGGACATGAAACCCGTAACAATTATTGATTTTCTTCTCCACGGGATATTTCCTTGT
GTTCGCGAAGACGCATATTGCAATGAAGACATTGACAGAGTTATGAATGTTGTTATCAAGAGATACGATATTCATAGC
GGTAACCGAGCGAGATTAGAAGAAGAGATGAGAGATCTGGCCTTTGTCCCGACCAAACATCGTCGTGTTAAAGCAAGG
GAAATCTTTGATCCAAGGAATGAACGTCTTTGTCACATTTTCGCAGAAGAAGACGTCTTTCCAATTGGAGAGCAATAC
AATGATCCGACAGTCCTCGCAGTTCTGCAGAAACTTGGAATGAAGAGCGAAAATGAAATAACTGCTCAAGATCTCTTC
CAAAGTACCCGCACAGTTTCCGAAATTATCAACAAGAGAAGAGCAGAAGTCAAGTCGGAAGCAATAATGGTATACCTG
GAGAGTTATCCTGACAAGCTTGAAGAGCCAATAAATGGAAAAGAATTAGGGGATATACTTCACGAGACTTCTTGGATA
TCCAGAATTAATCAGACGCCTGACAGGTTTCCCAAGAGCCTCCCWTTCATTGGCGCGACCGAAGTAAAGCCCAGGTTT
TACAAGCCTGCAGAAATTCACAGTGCAGATTTTGTTGACATTATTGGATCCGTGAGACCGATTGTCAAAGTTCAGTCT
TGTGGGAAAGTAGCCAAGCATTTTGGCTGGGATAAAAAGCCACAAGTCACCGTAGTTGTGAAGCATTTGAAACTCGTA
ATTGACTCTTACAGTCAACAAGAAAAGTCGCTTTACATGATGATGGTGGCAAAGCTATACTCTTTCCTCGTTGATGCC
GAATACGCTCTTGTCAGGAACTCCTTTGAAGAAATGAACATTGTAAGATGGATCTGGAATGGAGATGGCTTTTCGGCT
CCCTCAGAAATACTTGCTGAAAAATCACTCTTTGATTTATCTCCTTACATCCTGTCCCTTCCTCCAGAAATGAAACAA
TATCAGACATTCTTTGCAATGCATGGCCTAGCGGTTGAATGCRACGCCCACGTATTGTTTCGTGTTCTTCGATTGATG
AAAGAGAAATATGAGCAAAATAACCCTCCTGTTGAGGTCAATGATGTGAAGCGAGATTTGCAGCTGTCTATTAACATT

TTAAATGACTTAAAGAGTCGTAACATTGAACTCCCTTCTTTGGGCGAAGAAAATGTGCTTATTCCAACGTTTGTAGAA

GGAGATGAGTTTGTGAGGCTTGCACCAGCTGAAAGCTGTGTCTATTGTGAACGCGAGTGGCTGCAACAGGAAAATGAT

GAAGAGGAAGATGGTTATTTCTTCGTCCATCCGTCGGTTTCTAATAGCACAGCAGAGTTCTTCGGTATTCGAACATTG

GGGCACGTCATGCTCGATCCCGACGAGCTAGGAGTAGGCGAAGAATTTGGACAGGAAGAAAAACTTACCCGTAGACTA

AATAGGCTTCTGGAAGAATACACTGATGGGTTCGCCGTGCCAAAGGAGCTTATTCAAAATGCAGACGATGCAGGTGCA

ACGGAAATCAAGTTTCTTTATGATGAGCGTCAAAATGAGGATGCCCTAACTTGTCTCATTGACGACGGCATGAGAGAG

TGTCAAGGTGCTGCACTGTGGGTGTACAATGATGCGGAATTCCGCGATGAGGATTTTGAAAACTTGACAAAGTTAAGT

GGAGCGACGAAGGAACATAGCACAGAGAAAATTGGAAAATTTGGCCTTGGCTTTAATGCAGTGTACAATCTCACAGAT

GTCCCCATGTTGGTTAGCAGAAATTACTTCGTTATCCTTGATCCCCAYACCTTCTATTTGGGAAAGGCAATTAGAAAC

AAAAGCAAACCAGGCATGAAAATCGACCTGAATAAAAACGTGAAGAGACTGCGAACATTTCGCAATCAATTCAAACCG

TTTAATGGAATATTTGGTTGCGATCTTGAACTCAAGGAAGAAAGAAACTCGTATTCTGGTACATTGTTTCGTTTCCCC

CTTCGAACCAAGGAACAAGCTGTAAAAAGCGAAATCAAGCAACTTCACTATGACAGCAGCCAAGTGAAAGCACTATTG

CTGAAGTTCATTCTTGGAGCAAGATCACTGCTTCTCTTCACACAAAATATCCGTAAAGTTAGCATTCTTCATTTGCCA

CATAAAGGAAACCAACCGGATGTGATTTTTGAACTTACTAAGGAACTTTCAGAACATGGAATCTTGAAAGAGCTCTCT

GTGCCATTCAGATTGTCCCCTCCCGCTGAGAATTTAAGCGAAAATGATCAGCTCCTTCTAACACAGTGCAACTTTCTA

AAAGCTTCTTCTGAGTTTGTCAAGAGTACGAAGCTTTGCGAGAATTCACGATCTGATTTGCTACAATCAGCGTTTAAA

GTTAATGCAGTTAGCACCGTTTCGAATTACGGACGTCGTTTCTTTGGGGACAAAGAAAACTTACCTTCCGGTGCAGAA

AAATGGCTAGTCGTCTCATCCATGGGCACTGGTGAAGCAATGACATTTGCACAGGCACAGCAAAACAGAGGCCTTCTC

TCAGCTGCTGGAGTTGCTGTTCAATTGACAATTGAACCAACTTCAGTCTCAGCTCCCATTTGCCCTCCCAAATATCCA

GGAGCTGTATTTTGTTACTTACCGCTTCCAATCGAGAGTGGACTACCCGTTCACGTCAATGGCGCGTTTGCAGTAGCC

TCCAACAGACGTTCTCTAAAAGAGAAGACGGAAGATGACAAAGATTGCACTGGAGTAGAGTGGAACGACGTTCTCTTG

AAAGATTCGGTTTGTGCTGCGTATCTTGACCTCATCACACACCTTAAACCAGCCATAAACAAGCCTGGAAGTGGGTAT

CTGTTCCACTCTTTGTGGCCAAGGGACTGCAAAGTGCAAACAGCTTTGACGCCTCTTTCACGTTCTTTTTACGAAAAT

CTTGTTCACACGAACTTTCCATTTTTTTCAAATGGTACCAGAAGGGTTGGAATCAAAAACGTTGTCTTCTTTCATCCA

GAGTTTCGAAATGATGAACAAATAGGAAATACAGCATTTGAAGTGCTGAAGTTGTTAGTACGTGACGATAAAGCTGTT

ATTGATCTTCCGCTTGAAGTGCTTGACTCGTTTAAAAACTATGGTCTTTTTCAAGAAATCCAATCTGGACAATTTGAT

GGGATGAGGTTTTTCCRCGAATTGTTTTTCCCAAACATCTCCACTCTGCCTCAGCAGTTAAGAGACAACTTAGTCCTA

TACGCTCTGGATGACAAACAAAAGAAATTTGATGATTTGATGAAAGAGTATGAATGCATCCCTACATCCCCTAGGGGA

ATAAGWCTAAGACGTCCTCAGCATCTGGTCAGTCCGAAACGAGAAGTGGGTCTGCTGTTTTTGCCACAAGATGAAAGG

TTCCCGCATGGCACCCAAGAGACATTTCTGGGTCCTATAAGGATGTCGAATCTTGAGCGACTTGGAATGTTGACTGAT

GATATTCGTTGGCTGGAAATTGCAGAAAGGTCGGAGAGTATTTGGTCCTTAAATCAAGAAGATAGCACTGAAGCGAAA

AAGAGGTCAAAAACCTTGATCGAATTTTTGGACAAAAAGCTGAAGTGTGAAGAACCTCCACCACAAGAAATTAAAGAC

AGGATTCTCCGGGCAAAATTCCTTCCAGCTCTACAGAAACCACCGTCCTTCCCCCTCTCTTGGAAGGGAAATCAAATA

CGAGATGGAAACAGGCAAGTCCTTGTGGCGCCATCGGAAAGCTTTTTGAAGAAGGAAAAGTATTTAGTATGTTGCAGT

GAGGTCCTAATAGACCTTCACATACCAGAGGTTGTGCAAGAATTGTTGATGTTGAACAGCAAGAAGCCAACTGTAAAG

CATGTAATGACTCAACTTAACGAGGCAATTGCTGCTGTCTCAAGTAGCTCTGGATTTGAAGAATTGAGGAAGACTTGC

TTGAAATCTTACAAATATCTTCAAGAGGCTCTTGAGGATAACCAAGGAGAAATTTTGCATTTACTTAAAGAGAAAGAG

TTTATTTTGGTGGGAAGGAATTTCTGTAGTGCAAAACAAATTGCGTTCAAGTTGGACACAGATTGCTCACCTTATTTG

CAAAAGGTTCCTGATGAACTGGTCAGAACGTGTGGGAACTTTTTGAAAGCAGTTGGCGTAAAAGATGTTTTCTGTGCG

AAAGATTTCATTGATTCGTTGGAGTGCATTAAACACAAATTTGGAGAACAAGCGCTAGACAGGCAGTCGCTTCATGTC

GCTGTTCTTTTGGCAACACAATTAGAAAAATCCCTTGAGGATCATGAAGTTACCATTCCAACTGATAAGAGGAGAGAA

TCTATCTATCTCCCCAACTCTGATGGAGTAATGCAGCTGGTGAAGGATCTTTGTTTCAACGACTGCCAGTGGATCTCT

AAAGGTAACAGTGTTCAGTTTGTCAGTAGCAGAATTCCACACCCGACGGCTACCATGCTTGGAGTCAAAACTAAAAGG

CAAGAGGCTTTGCGAAAACGCACTCTCGGAATATCGTTTGGTCAAAGAGAGAAACTTACAAACCGCTTGAAACGTATT

CTCACTGGCTATCCCTGTGGTAAGGAACTATTGAAAGAAGTAGTGCAGAATGCAGATGACGCAATGGCAACAGAGGTC

TGTTTCGTTAAGGATCCTCGCACCCACCCAAAGAAATATATTTTTGACCCTTGCTGGGAGCCACTACAGGGTCCAGCA

TTGTGCGTGTATAACAACAAACCTTTCAAGAAGGTCGACATTGAGGGTATACAAAGTCTTGGACAGGGCAGTAAAGGT

GACGATCCAAACAAAACAGGCCAGTATGGCGTGGGCTTTAATGCTGTCTATCATTTGACAGATGTGCCTTCTTTCATG

TCTCAAGGAGAAGAGATTGGTGAAGTTTTGTGTGTGTTTGATCCACATTGTCGGTATGTACCTGACGCCAACCCTCAA

GAACCAGGAAGAATGTACAATACCGAGGACATGAAGGAAATGTTTTCAGATGTGTTTTCATGTTACCTAGGAGACTAT

TTTCCCCTCAGGAATTCAACAATGTTTAGATTTCCTCTTAGAACGCAAGCAATGGCAGACAACTCTCAGCTATCAGAA

ACGGAATTTACACTGAATGATCTGAGTAAATTAATGAAAGCACTGCAGAGGGAGCTTTTTGAGGTGCTTCTTTTCCTA

CACAGCGTGAAAAAGATCACGTTGTGCGACTTAGATGAGAGTGGAAATGTGTTGAATTCATACTCTGTGGAAGCAGTT

ATATCCCATGAGGATTCAGTAAAACAGGAGGAATTTAACAGGCACAGAAAGGAGATGGGAGAGGAGATGAAAGTAAGG

GGATTTCCYTCCAAAGCAGAGACGTATTCGTGCTCTTACGTAGTAACCTTAAACGACAGTTCTGGAAACAGGGAGGCG

TGGTTCATTGTTCAGCAGCTGGGGTTCGAAAACGATGTTCCAAAAAGTGTTGTTGAAGCCTACAGGAGACACGATCTT

GGGATGCTTCCTCTGGGAGGAGTTGCTTGTCTCTTGGAGCAGCAATCAAAAGGGAGAAAAGGTATGTTGGCTCAAGGA

AGGGCAAAAGCATTTTGCTTTCTGCCACTTCCCTTAGAAACCGGTCTTCCAGTGCACGTAAATGGTCACTTCGCACTG

GATCATGAAGCAAGACGACACATGTGGTGGGATGAAACTGGCGGATACCGAACTGAATGGAATGAAGTTTTGTTAAAA

GACGTAATAGCATCTTGCTATTTAAAGCTTTTGGCAGAGGTACGTAGCCTCTACCACCCCAAACTCATCCAAGGAGCA

GAGCCTCTGAACGGCCCCGAAGAAGTCCTAGTGAAAACACTTAAGGACTACGAGAATTTGTTTCCAAAA---

GTCAGTCCAGCGAAAGGTGATTATTGGGTGTTGCTTGTAAAATCCTTGTATCGGAAAATGAATGAAAAGCAGATGCGA

CTTCTTCCAGTGGTGAGGGAAGTACCAAAGAAGCAGCAAGTGCAAATAACGTGGCTCCCACCAAGAGGATCTGGAAAG

GACCAAGCTTTCTTCAATAATCTCAAATCTGATGAGAGACCCAAAGGAGAAAAGCAAGAAGAGATGTCTTTGTCAGAG

ATTTTGATTCAAATGGGTTTTAACCTCGTGCGTTTTTCGTTGTCTATTTGGGATGCGTTGAACAAATCTGGTGTAACT

GCCACCTGCATCTCGCCGTCATCAGTAATGACCTTTTTGAAGGGCTATAGTTCTCAAGATAGTCATTGTAGTATTGGA

TTCATACCAACTGATGTCAGCAATACGCCTTTGTTGACTGAAAGAGGCGTGGAAGAGGTGCTAAGATACTGCAAGGAT
GCTGAAGGCTTTTACGATGACTTATCTGGTCTCCCCTTACTTCTCACCCAAGACAACGTCCTTCGTGTGTTTGACGCA
AGAAATCCAACATTTTTATCGCGTTACCATGATATTTTGCCACAGAGTAAAGAGATGTTTGTTCATGATCGACTGCGA
AAGAATTTATTTCACGATCATAGAAGTCAGAATGCCCATGTGTTTCAACCCTTTGACGTAAAATGTTTTGCCAGTTAT
TTGCACCGTACACTACCGCTTGGATATTACGRCAGGAACGATTATGTGAGCTGGTCTCCAGATCAGCGTTCCGATGCT
GAGCCAAGTCGTCGTTGGATTTCCAGAGTATGGAGCTTTCTACATGAAATCGTGAAGGCTAAGAACTTTCTCGACATT
AACGATTCAAGCTGCATTCAGAGAGAACTAGAACCCTTGCTCAACTGGAGTATTCTTCCAGCGAAAACGCAAAGTACA
GCGTTGCAAGGTAGTAGCAGCTTAAACTTTATTGTCCCGCAAACAGCTAAAGTTCTGGTTCCCCTGAAATTGGCTAAG
TCAATTTTAGATTGCGCTTACTGGGAAATGGTCCCATTGCATAAGCCTCTGGTCCACGTCCTTCGAAAACTGAATCCC
CCCGAGCTTGATACTTCTGTTCTTGATGTCAGTCCTAACACGCATCCCGCGTGTCTTGCACGACATTTTATTGCATCA
TTAAAGATCCCGGAATCCCTCCTTTACGCACTTAATCATTGGATGATGACTCAGCCCAATTCATTGAAAGGTAARTTG
AGTCCAGACGACTGCAAGACATTGTTGAAATATTTCAGCGACAATGTAAACACCCTTCAGTCCACCAACGCCCAGCGA
GAAGTTTTAAAAAGGCTCCCATTTTATCTTGCAACGGACGGCGATCTTCTCAGTCTTGAAAAAAAGCAAACTTGCCTT
CTACCAAGTGGAATACCAAGAGTGGGAATAAGAACTTTACAAGGACAAGTCGATGTTCTTTTTCTAGAATCTGTGGAA
GGTCTCTCCAGCCTCTATACTTTTTTGAATTTTGATACAGTCTCTCAAGTCAGCGTTTACTGTGAGTTTATCTTCCCA
CATTTCAAGCTCCTCAGCAAGGAAGCAAGGGAAATGCACCTGAAGTATGTTCGAGATCATATTTTGGAAAGTTTTTTC
ACGACTAAAGATGACAAAATGAAAATTAGTAACTACTTGAAGACATCAGAAGTTGTCACCGTGGAGGATGGTTCCTTG
AAAAGAGCCAGTTGTTTTTACGACCCTTTTAACGATGTCTTTAGGATCATGCTTCCAGAAGATAAATTTCCACCGGAA
CCTTTCAGGTCAGGGTCGTGGTTGCCTTTCATGAAAAATATTGGAATGGTGTATGAAGTGTCTCCCGCCTTATTGAAG
AAATTTGCCGAGGACACCGCAAGAGAAGCCTCTTTGCAACGTACCTTGGAAACTGACAGAAAGTCTGAAGTGCTTGTT
TCATGTCTCTTCAAGCCACCTCATGTGAATGATGCAAGTCTTCTGCAAGCTGTAAGTGGAGTTCGCTTTGTAGTTTCA
GACTCCGTTCCTCCTAACCTGCTACAATTATATGCACAGTATAAAGATGAGCATAGCCATTACACTTCATTCAAGGAC
TCTATTGTTGCTGATTTTACTGAGGTTGTGTGGACAGTAGCACATCTTCTCCCGCGCTGGGCAGACCCGAGACCTCAC
AGTTTCCAAAACAACTTTCTCCTTTCGAGACTTCATGTTTTGTCACAGCCCAGCCCAGAAATGGTTATTTCTCACTGC
TCTAAGATATGTTGGCATTTGTCAAAACAGAATAACGACAAAATTTCCGACAGTGAAATTGCCACCAGAAAGTCAGTC
ATGCGAGAGGTGTACAAATTTTTCAGTGACAAAGTTAGTTCAAGTTATAACCTGAAGGAGAAGCTTGTAAACGTACCT
TGTATTTTGGTTGAAGATGGAAGGAGGTTCGTACAGGCGAAGCAAGTCGTGCTTGAGCTTCTCGAGAATGATGAGATA
CCTCCCTTTCTTTTCCGAGTGCCTTTCGAGTTTGGGGCGTATCACTTATGTTTTCAGAGTTTAGGGTGTTCAAAATCC
GTGAAAACGTCTCACTACGCGTTCCTTTTGGAAATGGTGTACAAGCAGTGCAATGGAAAAAAGCTCCTCCCCAAGGAA
GTTGAAAGCTCCTTAAAGGCAGTAAGAGGTCTCTTCGAAAGGCTTGAACATGATTCAGAGGAAGGCGTTCATCTCTCA
GATGTGTACTTGCCAGCAGTTCATCGATTCAACAGCGATTCGATTGGGAATCCGTCTGTAACCTTGCACAAGTCCACT
GATCTAATTTTCGATGACGCACCCCGGTATCGAAGCCGACTTGGAAACTTCAATGAATTGTTTGTGGTGGACCTCAAG
AGGACCGGATTGCAGAGCACGACATTGACGAACTACAGAGATTACATCATTCGCCTTCCAAGCGAATCTCGAACAAAG

ATGCTATCAGAGGAAGTGAAGGAAACGCTTGTCCACCCTACTGAACCTGTAAATGATAATGGCATAGCTGAGTCGTTA
AAAGAACAACTTTGTTCAGAGCAGTTTATTTATGGCATTGTAAGACTACTGCGCCACGCAAACCCGGACAAACCACAT
CTAGTCGAAACTGTCAGTACGACATTAGAAGCAAAACTGAGAAGCATCCAGATAATCGGAAGACCGAAAATTGAAACC
CACCTAATCCACGAAGGCCATCTTATACCAGGAAGCCAGACGGAAGTGGCATACTTTGTTGACAAGACATTAGATGGT
GACRAAGCCGTTTGGAAGCTCTACGTTAATGCAGAAGCAGAGGAAGATCGTGGTAAAATATGTTTGGTCTTAACCCAA
GTTATAGCCGAGGCATGTGAAGGGCTGCTACGCGATGCAGTGGCTTATGTCACAGAGATGTTACGCACAGACCCTGCC
AAGATATGGTCAGTATTAGACGACATGGAGATTCGCCAAGACGACTCTTACGATCCTTTAGATGGCAATGTGTTGCCT
CAACCAGGAAATTTCATTCCGATTGAAGACCATCACCTATTGAACGAAGCATTTGAAGAATTTTCACAAGGGGAATAT
GTTGGCTTAGAATTGGAGGATCCTAGTCTCGAGCAGAAGGATGGAGATGCTACTTTCATTTACGCTATCATAACTGGG
GAAGTAGAAGGCCAAAAGGATGCTACCCTGTACACAAAACTTTACCTGGTTAAAGTAGATCGCGACAGAGAACCCCAG
CAAAAGGAATCGGCTGATTTGTACAAATTTCATCGTATTCAATCAATTGATGAAGCCCACCAAGAGCGAACCGATTTC
TCAGTTGAACAGAAARCTACGATTTTGGCCCATATCACGGAGATGATGGAGAATACCTTTCGTTTGCCCGAGCATAGA
CGGAGGAAAATCATCAAGCGATTATGCTTACAATGGCATCCTGAGAAGAGTCGCAAAGATGAAGCGTTTTGCCATCAG
GTTTTGCAGCATTTAAAGAAAGAGGTGGCAAGATTGAAGCAGGTTGAGCTACAACGAACAGGCTCCGAAGCGGAAAGC
CGCCAAGAAAAGCCATACGAGGCTTTCTTTGACTTGTGGAGAAAACGTGCCCAGAGACATCATGCTTCACGACAGTCT
TACAGAGAACGTTATGAAGCTGCTGGTGGTGCATCAATGCTCCAGAGGAATAAGTCTGGAATTCCGCCGAGTTTTTCT
AAGAAGAACCCTCAGCCGGGTGAGGCAAGGCGCTGGTTGAGACAGGCCGAAGCCGATTTAAGAGCTGCCGGGAAGGAT
TGGATGACTAGGAATCCGTCCTACGAGTGGGCGTGCTTCAAGAGCCACCAGGCAGCAGAAAAGGCGCTCAAAGCTGCT
CAATATGCAGATGATGCTTTTAAGACTCATGCTCATAGTCTTAAGAGGATTGTTTCTGGATTGGGTGACTCAGAATTA
CTCGAATTAGCAAGTCAACTGGAGAACCTTGTGGTGGATTCTACGCGCATGCGCTACCCGGACCAATTATGTTATCCT
CAGATTCCTAACGACGTATACACTGGCGAGACGGCTGCGACGGCTCATGAATTAGCCAGAAAGATTGTGGATAAAGTC
AGGAAAAAGGTTGCAAAGTGGTAG

>Amil_CS11_consensus

ATGAGACTGAAAGAACCTTTGTTCAAATCACTGTGCAATAAGCAAGTCATCCATACCGAGGCATGTGGAGGGAAATGG

CTCTACGTCGGAGAAGCTATCTTTGACCGATTAGCAGAAAACGACCCGGTTGAGTTGCTTCGAAGCCTGCTTCTCAAA

GCCCATGAAAACATCGCCACAGTCCCCAGCCACGTTTTGCAATCGATTCAAGACTTTGAACAATTTATCACAGAAGTG

ACACCTTCACTTGTACGTTCGGTTCTCAAAACAATGTCATTTTTGTATGAAAGTCTTGGAAAAGAAGAGAAGCTGCAG

CTTCTCAAGTTTGTTCTCAAAGACGAATGCTTTTCTGAGCTCTCAGGCCTAACACTGTTGCCCGTGTCTGATGGAGAA

TTTCTACCCTTCTCAAATTCAGATGAAACCATTTTTATTTCATCGCGAAAGCATCCCCCCGGACTGATACCGACATTG

CGTCACCGCCTTCTGGACCAATCCTTGGAGAGTGAAACCTTGACAAAACTGAAAGCTGTTGCAAAAGCAGAATGCACT

CAGTTGAAACTTCTTGAGAAATGCCACATACCCTCTCTATTGCGAGAAGCCTTATCTGGGAAATTGACAAATGAGGAT

CTTCTCGACGGGTACCCGGTAGACCAGTGGCTCAAGTGCCTATGGGAGTACTTAGGACAACATTTTAAGACGCATGAA

GACCTSTCTCTGGTGAATAACTTACCTTTAGTACCAGTTGATCCATCGAAAGGTGCCTTGACAATGCTAGCAAACCCG

TCTAAAGTTGTTGTTAGATGTCTGGATGACCAACGCTTAGAGAAGAACGTGAGCTCAGTGTTAGAAAATTTCGGGGTG

ATTGTCTTGGAATCTTTGCCAGACTACTTGAAACACCATCCCTGTGTTCTAGATACATACGTGYATCGACCGTCAGTC

CACGGTGTTTTGCAAGCGATGGCAGTTTCGGCTTCTGATTGTCTAGGAATGCTTTCAGCAGTCTTGCTGGACATGCAA

GATAAAGGTAGAGGTGACGACGTACTTTCACTGAGGAAATTTATTTCCAAGTCTGAGTCACTGGAGCCCAGAGAAAAA

GAAATTATTAGCTCGCTCCCACTTTTTGAGGAAACTGGACAGCCACATTCCTTTGTATCAAAGAAAGACGTTTGGGGT

GCTGCACCACAAGATGTAGACGACTATTTGGTGGCTCTCCCCACTGCAGCCAAATTTATTGACACAAGAGCTGACGAC

GCGAGACGGTTAGTTTCTTTGCTGGACATGAAACCCRTAACAATTATTGATTTTCTTCTCCACGGGATATTTCCTTGT

GTTCGCGAAGACGCATATTGCGGTGAAGAYATTGACAAAGTYATGAATGTTGTTATCAGGAGATACGATATTCATAGC

GGTAACCGAGTGAGATTACAAGAAGAGATGAGAGATCTGGCCTTTGTCCCCACCAAACATCGACGTGTTAAAGCAAGG

GAAATCTTTGATCCAAGGAATGAACGTCTTTGTCACATTTTCGCAGAAGAAAACGTGTTTCCAATTGGAGAGCAATAC

AATGATCCGACAGTCCTCGCAGTTCTGCAGAAACTTGGTATGAAGAGCGAAAATGAAATAACTGCTCAAGATCTCTTC

CAAAGTACCCGCACAGTGTCCGAGATTCCCAACAAGAGAAGAGCAGAAGTTAAGTCGGAAGCAATAATGGCATACTTG

GAGAGTTATCCGAACAAGCTTGAAGAGCCAATAGATGGAAAAGAATTAGGGGATGTACTTCACGAAACYTGTTGGATA

TCCAGAATTAATCAGACGCCTGACAGGTTTCCCAAGAGCCTCCCTTTCACTGGCACGGCCGAAGTAAAGCCCATTTTT

TACAAGCCTTCAGAAATTCACAGTGCAGATTTTGTTGACCTAATTGGATCCGTTAGACCGATTGTCAAAGTTCAGTCT

TGTGGGAAAGTAGCCAAGCATTTTGGCTGGGATAAAAAGCCACAAGTCACCTTAGTTGTGAAGCATTTGAAACTCGTA

ATTGACTGTTACAGTCAACAAGAAAAGTCGCTATACATGATGWTGGTTGCAAAGCTATACTCTTTCCTCGTTGATGCC

GAACACGCTCTTGTCAGGAACTCCTTTGAAGAAATGAACATTGTAAGATGGATCTGGAATGGAGATGGCTTTTCGGCT

CCCTTAGAAATACTTGCTGAAAAACCACTCTTTGATTTATCTCCTTACATCCTGTCCCTTCCTCCAGAAATGAAACAA

TATCAGACATTGTTTGCAATGCATGGCCTAGCGGTTGAATGCGAAGCCAACGTATTGTTCCGTGTTCTTCGATTGATG

AAAGAGAAATATGAGCAAAACAACCCTCCTATTGAGGTCAATGATGTGAAGCGAGATTTGCAGCTGTCTATTAACATT

TTAAATGACTTAAGGAGTCGTAACATTGAACTCCCTTCTTTGGGCGAAGAAAGTGTGCTTATTCCAACGTTTGTAGAA

GGAGATGAGTTTGTGAGGCTTGCACCAGCTGAAAGCTGTGTCTATTGTGAACGCGAGTGGCTGCAACAGGAAAATGAT

GAAGAGGAAGATGGTTATTTCTTCGTCCATCCGTCGGTTTCTAATAGCACAGCAGAGTTTTTCGGTATTCGAACAYTG

GGGCACGTCATGCTCGATCCCGACGAGCTAGGAGTAGGCGAAGAATTTGGACAGGAAGAAAAACTTACCCGTAGACTG

AATAGGCTTCTGGAAGAATACRCTGATGGGTTTGCCGTGCCAAAGGAGCTTATTCAAAATGCAGACGATGCAGGTGCA

ACGGAAATCAAGTTTCTTTATGATGAGCGTCAAAATGAGGATGCCCTAACTTGTCTTATTGACGACGGCATGAGAGAA

TGTCAAGGTGCTGCACTGTGGGTGTACAATGATGCGGAATTCCGCGATGAGGATTTTGAAAACTTGACAAAGTTAAGT

GGAGCGACGAAGGAACATAGCACAGAGAAAATAGGAAAATTTGGCCTTGGCTTTAACGCAGTGTACAATCTCACAGAT

GTCCCCATGTTGGTTAGCAGAAATTACTTCGTTATCCTTGATCCCCACACCTTCTATTTGGGAAAGGCAATTAGAAAC

AAAAGCAAACCAGGCATGAAAATCGACCTGAATAAAAACGTGAAGAGACTGCGAACATTTCGCAATCAATTCAAACCG

TTTAATGGAATATTTGGTTGTGATCTTGAACTCAAGGAAGAAAGAAACTCATATTCTGGTACATTGTTTCGTTTCCCC

CTTCGAACCAAGGAACAAGCTGTAAAAAGCGAAATCAAGCAACTTCACTATGACAGCAGCCAAGTGAAAGCACTATTG

CTGAAGTTCATTCTTGGAGCAAGATCACTGCTTCTCTTCACACAAAATATCCGTAAAGTTAGCATTCTTCATTTGCCA

YACAAAGGAAACCAACCGGATGTGATCTTTGAACTTACTAAGGAACTTTCAGAACATGGAATCTTGGAAAAGCTCTCT

GTGCCATTCAGATTGTCCCCTCCCGCTGAGAATTTGAGCGAAAATGATCAGCTCCTTCTAACACAGTGCAACTTTCTA

AAAGCTTCTTCKGAGTTTGTCAAGAGTACGGAGCTTTGCGAGAATTCACGATCTGATTTGCTACAATCAGCCTTTAAA

GTTAATATAGTTAGCACCGTTTCGAATTACGGACGTCGTTTCTTTGGGGACAAAGAAAACTTACCTTCCGGTGCAGAA

AAATGGCTAGTCGTCTCATCCATGGGCACTGGTGAAGCAATGAAATTTGCACAG------

CAAAGCAGAGGCCTTCTCCCAGCTGCTGGAGTTGCGGTTCAATTGACAATTGAACCAACTTCAGTCTCAGCTCCCYTT

TGCCCTCCCAAATATCAAGGAGCTGTATTTTGTTACTTACCGCTTCCAATCGAGAGTGGACTACCCGTTCACGTCAAT

GGCGCGTTTGCAGTAGCCTCCAACAGACGTTCTCTAAAAGAGAAGACGGAAGATGACAAAGGTTGCACTGGAGTAGAG

TGGAACGACGTTCTCTTGAAAGATTCGGTTTGTGCTGCGTATCGTGACCTCATCACACACCTTAAACCAGCCATAAAC

AAGCCTGGAAGTGGGTATCTATTCCACTCTTTGTGGCCAAGGGACTGYAAAGTGCAAACAGCTTTGACGCCTCTTTCA

CGTTCTTTTTACGAAAATCTTGTTCACAGGAACTTTGCATTTTTTTCAAATGGTACCAGATGGGTTGGAGTCAAAAAC

ATTGTCTTCCTTCATCCAGAGTTTCGAAATGATGAACAAATAGGAAATACAGCATTTGAAKTGCTGAAGTTGTTAGTA

CGTGGCGATAAAGCTGTTATTGATCTTCCGCTTGAAGTGCTTGACTCGWTTAAAAAGTATGGTCTTTTTCAAGAAATC

CAATCTGGACAATTTGATGGRATGAGGTTTTTCCGCGAATTGTTTTTCCCAAACATCTCTACTCTGCCTCAGCAGYTA

AGAGACAGCTTAGTCCTGTACGCTCTGGATGACAAACAAAGGAAATTTGATGATTTGATGAAAGAGTATGAATGCATC

CCTACATCCCCTAGGGGAATAAGACTAAGACGTCCTCGGCATCTGATCAGTCCGAAACGAGAAGCGGGTCTGCTGTTT

TTGCCACAAGATGAAAGGTTCCCGCATGGCACCCAAGAGACGTTTCTGGATCCTTTAAGGATGTCGAATCTTGAGCGA

CTTGGAATGTGGACTGATGATATTCCTTGGCTGGAAATTGCAGAAAGGTCGGAGAGTATTTGGTCCTTAAATCAAGAA

GACAGCACTGAAGCGAAAAAGAGGTCAAAAACCTTGATCGAATATTTGGACAAAAAGCTGAAGTGTGAAGAACCACCA

CAACAAATTAAAGACAGGATTCTCCGGGCAAAATTCCTTCCAGCTCTACAGAAACCACAGTCCTTCCCCCTCTCTTGG
AAGGGAAATCAAATACGAGATGGAAACAGGCAAGTCATTGTGGCGCCATCGGAAAGCTTTTTGAAGAAGGAAAAGTAT
TTAGTGTGTTGCAGTGAGGTCCTAATAGACCTTCACATACCAGAGGTTGTTCAAGAATTGTTGATGTTGAACAGCAAG
AAGCCAACTGTAAAGCATGTAATGGYTCAACTTAACGAGGCAATTAATGCTGTCTCAAGTGGCTCTGGATCTGAAGAA
TTGAGGAAGACTTGCTTGAAATCCTACAAATATCTACAAGAGGCTCTTAAAGATAATCAAGGAGAAATTTTGCATTTA
CTTAAAGAGAAAGAGTTCATTTTGGTGGGAAGGAATTTCTGTAGTGCAAAACAAATTGCGTTCAATTTGGACACAGAT
TGCTCACCTTATTTGCAAAAGGTTCCTGATGAACTAGTCAGAACGTGTTGGAACTTTTTGAAAGCAGTTGGCGTTAGA
GATGTTTTCTGTGCGAAAGATTTCATTGATTCTTTGGAGTGCATTAAGCACGAATTTGGAGAACAAGAGCTAGACAGA
CAGTCGCTTCTTGTCGCTGTTCGTTTGGCAACACAATTAGAAAAATCCCTTGAAGATCATGAAGTTACCATTCCTACT
GATAAGAAGAGAAAATCTATCTATCTCCCCAACTCTGATGGAGTGATGCAGCTGGTGAAGGATCTTTGTTTCAACGAC
TGCCAATGGATCTCTAAAGTTAATAATGTCAAGTTAGTCAGTCCAAAAATCCCACCCTCAACAGCTACCATGCTTGGA
GTCAAAACTTTAAGGCAAGAAGCTTTGCAAAAACAGACTCTCGGGATATCGTTTGGTCAAAGAGAGAAACTTACAAAC
CGCTTGAAACGCATTCTCACTGGCTATCCCTGTGGTAAGGAACTATTAAAAGAACTAGTGCAGAATGCAGATGACGCA
ATGGCAACAGAGGTTTGTTTTGTTAAGGATCCTCGCACCCACCGAAAGAAATACATTTTTGACCCTTGCTGGGAGCCA
CTACAGGGTCCAGCATTGTGCGTGTACAACAACAAACCTTTTAAGAAGGTCGACATTGAGGGTATACAAAATCTAGGA
CAGGGCAGTAAAGGTGACGATCCAAACAAAACAGGCCAGTATGGCGTGGGCTTTAATGCGGTCTATCATTTGACAGAT
GTGCCTTCTTTCATGTCTCAAGGAGAGGAGATTGGTGAAGTTTTATGTGTGTTTGATCCACATTGTCAGTATGTACCT
GACGCCAACCCTCAAGAACCAGGAAGAATGTACAATACCGAGGACATGAAGGAAATGTTTCCAGATGTATTTTCATGT
TACCTAGGAGACTATTTTCCCCTCAGGAATTCAACAATGTTTAGATTTCCGCTTAGAACGCAAGCAATGGCGGACAAC
TCTCAGCTATCAGAAACCGAATTTACACTGAATGATCTGAGTAAATTAATGAAAGCACTGCAGAGTGAGCTTTTTGAG
GTGCTTCTTTTCCTACACAGCGTGAAAAAGATCACGTTGTGCGACTTAGATGAGAGTGGGAATGTGGTGAATACGTAC
TCCGTGGAAGCAGTAATGTCCGATGAGGATTCAGTAAAACAGGAGGAATTTAACAGTCACAGAAAGGAGATGGGAGAG
GAGATGAAAATAAGTGGATTTCCCACCAAAGTAGAGACGTGCTCATGCTCTTACGTAATAAACTTACACGATAGTTTG
GGAAACAGGGAGACATGGTTCATTGTTCAGCAGCTGGGGTTTGAAAACAATGTTCCAAAAAGTGTTGTTGAAGCTTAC
AAGAGACACGATCTTGGAATGCTTCCTCTGGGAGGAGTTGCTTGTCTCTTGGAGCAGCCATCAAGAGGGAGAAAAAAG
ATGTTAGCCCACAGCAAGAAAAAAGCATTTTGCTTTCTGCCACTTCCCGTAGCAACTGGTCTTCCAGTACACATAAAT
GGTCACTTCGCACTAGACCATGAAGCAAGACGAAACATGTGGCGGGATGAAACGGGTGGATACCGAACGGACTGGAAT
GAAGCCTTGTTAAATGACATAATAGCACATTGCTATTTAAAGCTCTTAGCAGAGGTGCGTAGCCTGTACCGTCCCAGC
CTCGTCCAGGGAGCAGTACCTCTGAATGGTGCCGAACAGWATCTAGTGGAAACACTTGAGGACTACGAGAATTTGTTT
CCAAAAAGAGTCAGTCAAGCGAACGATGATTATTGGGTGTTGCTTGTAAAATCCTTGTATCGAAAAATGAATGAAAAG
CAGATGCGCCTCCTTCCAGTAGTGAGGGATGAACCAAACAAGCAGCAAGTGGAAATAACGTGGCTCCCACCAGGAGGG
TCTGGAAAGGACCAAGCTTTCTTCAATAATCTCAAATCTGATAGGAGATCCAAAGAAGACAACCACGAAGAGAAGTCT
TTGTCAGAGATTTTGATTCAATTGGGTTTCAACCTCGTGCGKTTTTCGTTATCTATTTGGGATGCGTTGATCGAATCT

GGTGTAACTGCCAGCTGTGTATCACCTTCGTCGGTGATGACTTTTCTAAAGGGCCATAGTTGTCAAGATAGTCATTGC

AGTATTGGATTCATACCAACTGATGTCAGCAATACGCCTTTGTTGACTGRAAAAGGCGTTGAAAAGGTGCTACGATAC

TGCAAGGACGCTAAAGGCTTCCTCGATAACTTATCTGGTCTCCCCTTACTTCTTACCCAAGACAACGTCCTTCGCGTG

TTTGACTCAAGTAGTCCCAGATTTTTATCGCAGTACCATGACATTTTGCCACAGTGTAAGGAGATGTTTGTTCACGAT

CGACTGAGACTGAGTATATTTCATGATCAAAGAAGTAAGCTTGCCTATGTGTTTAAACCCTTTAACGTTAAAGGTTTT

GCCACATATTTGCATCACACACTGCCACCAKYATATCGCGGAAGAACTAATTATGTTAGCTGGTGTCCAGATAAGCGT

TCCGATGCTGAGCCAAGTCGACTTTGGATTTTCAGAGTATGGAGCTTTCTAAATGAARTYGTGACGGCTAAG---

GAGTTAGAGATGGGAGGYTCAAGATGCATTCAGGAAGAGCTAGAACCCTTGCTCAGTTGGAGTATTCTTCCAGGGAAA

ACGCAAAGTCCAGCGTTGCAAGTTAGTAGCATCTTAGACTTTACTATCCCGCAGACAGCTAGAGTTCTGGTTCCCTTG

AAGTTGGGTAAATCAATTCTAGATTGCACTTACTGGGAAATGGCCCTAATGCATAAGCCTCTTGTCCGCGTACTCCGA

AAGCTGAGTCCTCCCGAGCTTGATACATCTGTTTTCGGTGACAGTTCGTACACGCAGCTTGCGTCTCTGGCACGAAAT

TTCATCGCCTCGTTAGAAATTCCGGAATCCCTCCTCTACGCTCTTAATCATTGGATGACMACTCATCCCAACTGTCTG

AGGGATAAATTGAGTCCTGACGACTGCAGGACATTACTGAAATATTTCAGTGAAAATGCAAACACCCTGAAGTCTACC

AACGCTGAGAGGGAAATCTTAAGAAGGCTCCCATTTTATCTTTCGACCCACGGCAATCTCATCAGTCTTGACAACAAG

CCAATTCGCCTTCTACCGAGTGGAATCCCAAGAGATGAAATAAGCATTTTACAAAGGGAAGTCAATGTTCTTTTTCTG

GAATGTGTTGAAGGTCTCTCCAACCTTTATGACTTTTTGAATTTTGATACAGTCTCTCGAGTTAACGTGTACTGTGAG

TTCATCTTCCCACACTTCAAGCTCTTTAGCAAGGAAGCAAGAGAAATGCATCTGAAATATGTTGGAGAAGTTGTTTTA

GCAAGTATTTTCACGACTKATGATGACAAATTGAAAATAATAAACTGTCTAAGGATATCGGAAGTTGTCACCCWAGAG

GATGGTTCTTTGAAAACAGCCGATTGTTTTTACGACCCTTTAAACGATATATTTCAGATTATGCTTCCAGAAGACAAA

TTTCCACCGTACCCTTACAGGTCATCGTTGTGGTTGCCTTTTATGAAGAAGATTGGAATGGTGCATGAAGTGTCTCCC

ACCTTATTCAAGAAATTTGCCAAGGACATTGCTAGAGAAGCCTCTGTGCAACCTACCGAGAAAACAGACCGAAAGTCT

AAAGTGCTTGTTTCGCGTCTGTTCACCCCACCACATGTGAATAATKCAAGTCTTCTGCAAGATATTAGTGGARTTCGC

TTCGTGATGTCAGATTCTGTTCGTTCTGACCTGCTACAATTRCATTCACAGTATGAAGATCAGCATAGCCATTACATT

TCATTCAAKGACTCTGTTGTTGATGAATTTACCGAGACTGTGTGGACAGTAGCACATCTTCTCCCGCATTGGGCGCAC

CCGAGATCTCACGGTCTCCAAGACAACTTTCTCCTTTCAAAACTTAATGTTTTGTCAAAGCCTAGCCCRGAAATGGTC

ATTTCACACTGCTCTGTTTTATGTTGTCATTTGGCAAAACAAAACGGTGACATGATTTCYGAAARTGAAATTGCCACC

AGAAAGTCAGTCATGRGAGAGGTTTACAAATTTTTCAATGAGAACCTTGGTTCAAGTTATGACCTGAAGGAGAAGCTC

GAAAACGTACCGTGCATCTTGGTTGAAGATGGAAGGAGGTTTGTGCTGGCTAAGCAAACTGTGCTTAACCTTCTCGAG

GAGGATGAGATACCTCCCTTTCTTTTCCGAGTGCCTATCGAATTTGGAGCCTATCACTTATTTTTTCAGAATMTCGGG

TGTTCAAAATCCGCAGAAACGTCTCACTACGCGTTGGTGTTAGAAATGGTGTACAAKCAGTSCAATGGAAAAAAGCTC

CTTCCCATGGAAGTTGAAAGCTCCTTGAAKGCAGTAAGAGGTCTCTTTGAAAGGCTCGAAGGTGTTTCAGGGGAGGAC

GTTCCTCTCYCAAGTGTGTACTTGCCAGCWGTTTATCGATTCAACAACGGTTCCATCGGGAATCCATCTGCAACCTTG

CACAAGTCCACTGATCTCATTTTCGATGACGCACCCCGGTATCGAAGCCGAATTGGAAATTTCAATGAATTGTTTGTA

GTGGACCTAAAGAAGACGRGACTTCAGTGCACAACATTGACGAACTACAGAGATTACATCGTCCTCCTTCCAAGCGAA
TCTCGACCAAAGATGCTATCAGATGAAGTGAAGGAAACGCTCGTTCAYCAGCTTGATTCTGAAAATGGTGATTGCATA
GCTGAGTCATTAAAAGCGCAACTTTGTTCAGAGCARTTTATTTATGGAATTTTAAGGCTAATGCGCCACGCAAACCTG
GACAAACCACATCTGGTCGAAACTGTCAGTACTACATTAGAAGCAAAACTGAGAAGCATCCGGATAATSGGAAGACCG
AAAATTGAAACCCATCTAATACACAAAGGCGATCTTATACCAGGAAGCCAGATGGAAGTGGCCTACTTTGTTGACAAG
ACAKTAGATGGAGACGAAGCCGTGTGGAAGCTGTACATCAATGCAGAAGCAGAAGAAGATCATAGTAAAATATGTTTG
GCCTTAACCCAAGTTATAGCCGAGGCATGTGAAGGGCTGCTACKCGATGCAGTGGCTTATGTCACAGAGATGTTACGC
ACAGACCCCGCCAAGATATGGTCAGTATTAGACGACATGGGAATTCGCSAAGACGACTCTTACGATCCTTTAGATGGC
AAWTTGTTACCTCAACCGGGAAATTTTATTCCAATTGAAGACCATCACCTATTGAACGAAGCATTTGAAGAGTTTTCT
CAAGGGGAATATGTTGGCTTAGAATTGGAGGATCCTAGTTTCGAGCAGAAGGATGGAGATGCTACCTTCATTTACGCT
ATCATAACTGGGGAAGTAGAAGGCCAAAAGGATGCTACCCTGTATACTAAACTTTATCTTGTCAAAGTAGATCGCGAC
AGAGAACCCCAGCCAAAGGAATCAGCTGATTTATACAAATTTCATCGTATTCAATCAATT---
GAAGCCTACGAAGAGCGAACCTACTTCTCAAGTGAACAGAAATCTGAAATTTTGGCGCATGTAACTGAGATGTTGGAG
AATACTTTTCGTTTGCCCGAGCCGAGGCGAAGGAAAATCATCAAGCGATTATGCCTACAATGGCATCCTGAGAAGAGT
CGCAAAGACGAAGCATTTTGCCACCAGGTTTTGCAGCATTTAAAGAATGAGGTGGCAAGATTGAAGCAGGTTGAGCTA
CAACGAACAGGCTCCGAATCTGAAAGCAGCCAAGAAAAGGCATACGAGGCTTTCTTTAACTTGTGGACAGCACGTGCC
CGTAGACATCATGCTTCAAGACAGTCTTACAGAAAACATTATGAAGCTTCTGGTGGTGCATCAATAATCCAGAGAAGG
AAGTCTGAAATCCCGCCGAGTTTTTCTACGAAGAACCCTCAGCCGGGTGAGGCAAGCCGCTGGTTTAGACAGGCTGAA
GCCGATTTAAGAGCTGCCCAGAAGGATTTGATGACTGGCAATCCGTCCTACGAGTGGGCGTGCTTTAAGAGCCACCAG
GCAGCAGAAAAGGCGCTCAAAGCTGCTCAATTTGCAGATGATGCTTTCAGGACTCGTGCTCATAGTCTTGTGAGGATT
GTTTTTGGTTTGGGTGACTCAGAATTACTCGAATTAGCAAGGCAACTGGAGAACCTTGTGGTGGATTCTACGCGCATG
CGCTACCCGGACAGAGTCTCCTATCCTCAGATTCCTAACGAGGTATACAGTGGCGAGACGGCCGAGACGGCTTATGAA
TTAGCCAGAAAGATTGTGGATAAAGTCAGGGAGAAAGTTGCAAAGTTGTAG

2 Description of the script

```
#! perl

SET_UP:
chdir;
chdir"desktop";
opendir(ANALYZE,analysis) or die "Make [analysis] folder on desktop.";
@files = readdir(ANALYZE);
closedir(ANALYZE);
@seq_files = grep(/.txt/,@files);
push(@seq_files,grep(/.nuc/,@files));
chdir"analysis";

MAIN:
foreach $file_name (@seq_files) {
 open(SEQUENCE,$file_name) or die "Unable to open file.";
 $file_title = substr($file_name,0,length($file_name) - 4);

 open (ALLELE1,">" . $file_title . "_allele1.txt") or die "Unable to make output file.";
 open (ALLELE2,">" . $file_title . "_allele2.txt") or die "Unable to make output file.";

 $search[1] = getc(SEQUENCE);
 $search[2] = getc(SEQUENCE);
 $search[3] = getc(SEQUENCE);
 $search[4] = getc(SEQUENCE);
 $search[5] = getc(SEQUENCE);
 $search[6] = getc(SEQUENCE);

START:
 $check = $search[1] . $search[2] . $search[3] . $search[4] . $search[5] . $search[6];
```

```
 if (length($check) <= 0) {
  seek(SEQUENCE,0,0);
  goto SCAN;
 }
 if ($check eq "ORIGIN") {goto SCAN;}

 $search[1] = $search[2];
 $search[2] = $search[3];
 $search[3] = $search[4];
 $search[4] = $search[5];
 $search[5] = $search[6];
 $search[6] = getc(SEQUENCE);
 goto START;

SCAN:
 $seq = uc(getc(SEQUENCE));
 if (length($seq) <= 0) {goto CLEAN_UP;}
 if ($seq eq "Y") {
  print ALLELE1 "C";
  print ALLELE2 "T";
  goto SCAN;
 }
 if ($seq eq "W") {
  print ALLELE1 "A";
  print ALLELE2 "T";
  goto SCAN;
 }
 if ($seq eq "S") {
  print ALLELE1 "C";
  print ALLELE2 "G";
  goto SCAN;
 }
 if ($seq eq "R") {
```

```
  print ALLELE1 "A";
  print ALLELE2 "G";
  goto SCAN;
 }
 if ($seq eq "M") {
  print ALLELE1 "A";
  print ALLELE2 "C";
  goto SCAN;
 }
 if ($seq eq "K") {
  print ALLELE1 "G";
  print ALLELE2 "T";
  goto SCAN;
 }
 print ALLELE1 $seq;
 print ALLELE2 $seq;
 goto SCAN;

CLEAN_UP:
 print $file_name . "completed.
";
 close(SEQUENCE);
 close(ALLELE1);
 close(ALLELE2);
}
```

3 Supporting Figures

Figure S1 Modified genetic structure of the sacsin-like gene. (a) The genomic location of the sacsin-like gene coding sequence (Amillepora11972-11974) in *A. millepora* genome assembly version 2.0.1 is shown with yellow boxes on chromosome 7. The RNA-seq reads mapped across the chromosome are shown under the structure of genes. The modified CDS is shown with blue boxes. (b) The locations of two incorrect insertions and two incorrect deletions in the reference sequence are indicated by black allows. Genomic DNA mapped reads (SRR10571533) and RNA-seq mapped reads (SRR2086157) on the up- and downstream of these indels are shown.

(a)

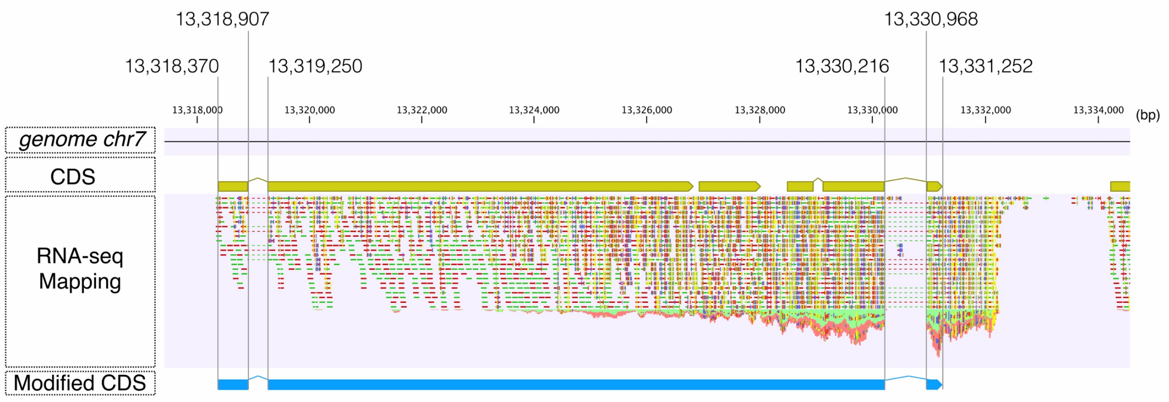

(b)

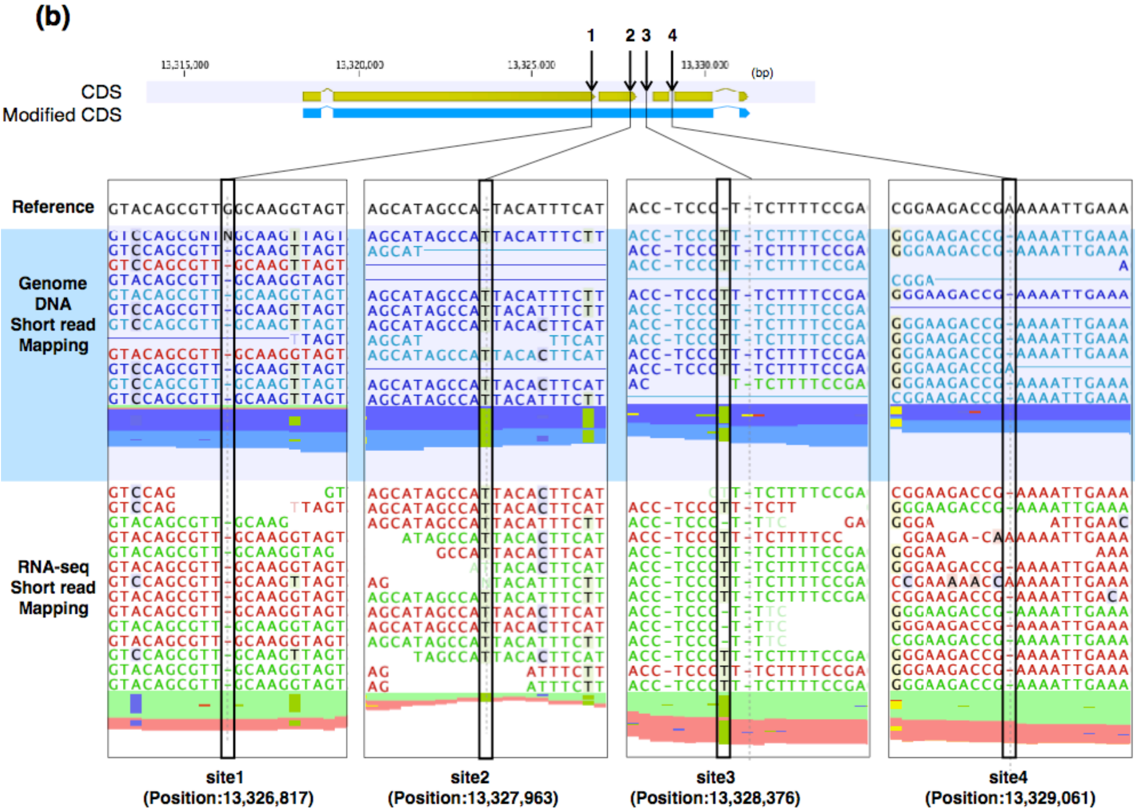

1
2 3
4
13,315,000
13,320,000
13,325,000
13,330,000
(bp)
CDS
Modified CDS
Reference
GTACAGCGTTGGCAAGGTAGT
AGCATAGCCA-TACATTTCAT
ACC-TCCC-T-TCTTTTCCGA
CGGAAGACCGAAAATTGAAA
Genome DNA Short read Mapping
RNA-seq Short read Mapping
site1
(Position:13,326,817)
site2
(Position:13,327,963)
site3
(Position:13,328,376)
site4
(Position:13,329,061)

Figure S2 Positions of primers for the examination of inversions upstream and downstream of the sacsin-like gene. Values of nucleotide diversity are shown at the top. The CDS of the sacsin-like gene based on the reference genome and the flanking downstream gene are shown in the middle. The assumed structures of the three different inversions are shown at the bottom of (a), (b), and (c). Positions of primers are indicated by arrows below the gene structure of the sacsin-like gene. Under the assumed inversion conditions, the primers included in the inverted region are marked in red. Directions of the arrows indicate directionality of primers (5′ to 3′). Targeted regions of each primer set are shown by white boxes.

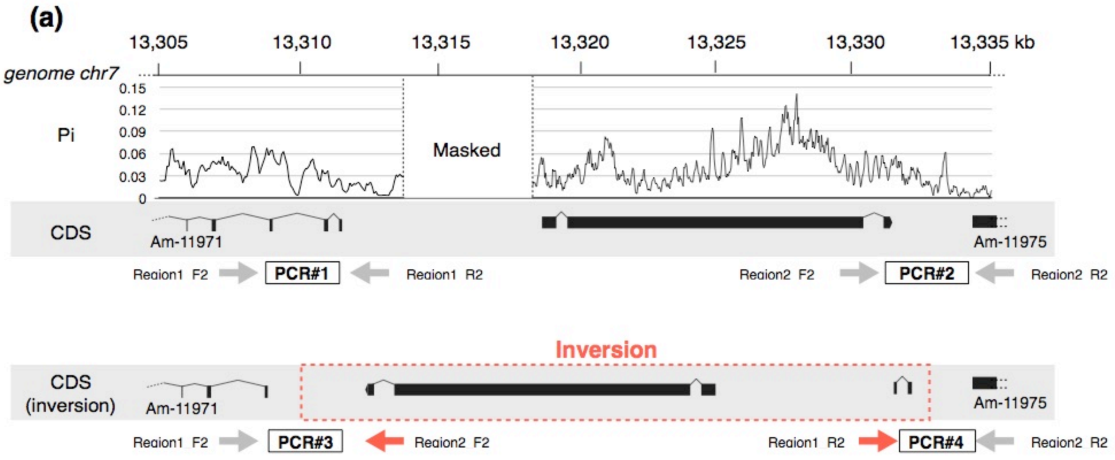

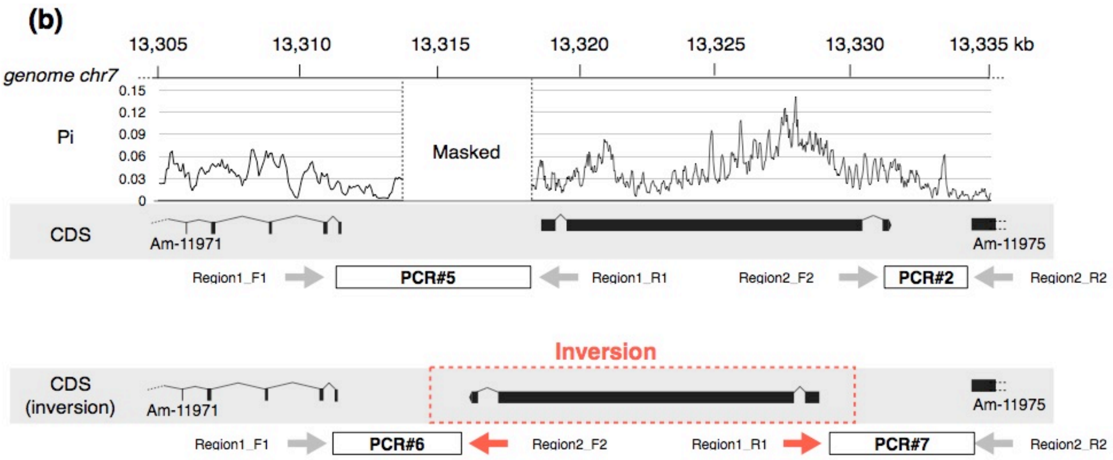
(b)
13,305
13,310
13,315
13,320
13,325
13,330
13,335 kb
genome chr7
0.15
0.12
0.09
0.06
0.03
0
Pi
Masked
CDS
Am-11971
Am-11975
Region1_F1
PCR#5
Region1_R1
Region2_F2
PCR#2
Region2_R2
Inversion
CDS (inversion)
Am-11971
Am-11975
Region1_F1
PCR#6
Region2_F2
Region1_R1
PCR#7
Region2_R2

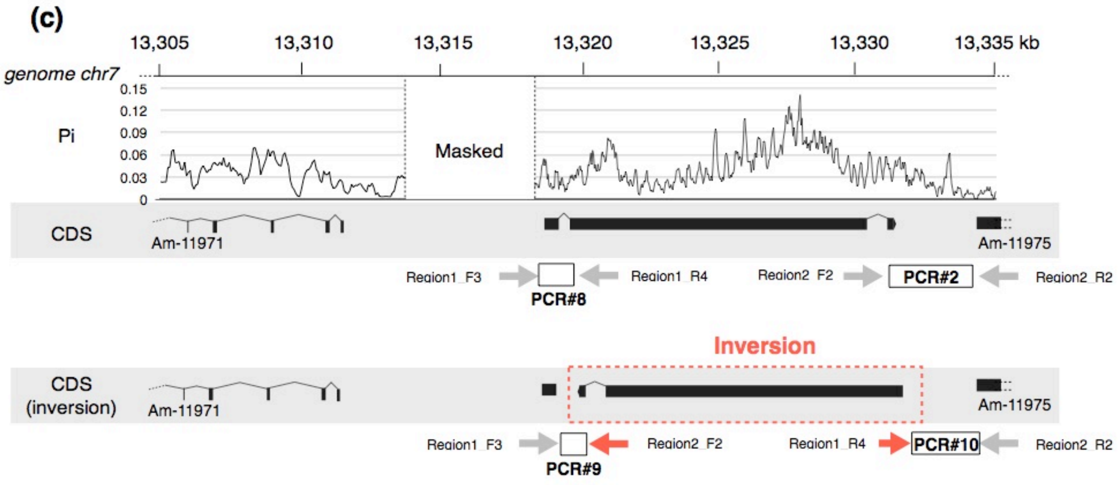

(c)
13,305
13,310
13,315
13,320
13,325
13,330
13,335 kb
genome chr7
Pi
0.15
0.12
0.09
0.06
0.03
0
Masked
CDS
Am-11971
Am-11975
Region1_F3
PCR#8
Region1_R4
Region2_F2
PCR#2
Region2_R2
Inversion
CDS
(inversion)
Am-11971
Am-11975
Region1_F3
PCR#9
Region2_F2
Region1_R4
PCR#10
Region2_R2

Figure S3 Electrophoresis patterns of PCR products. Four primer sets (PCR#1, 2, 5, 8: shown in black) are able to amplify the target sequence when the template genomic DNA does not contain an inversion of the sacsin-like gene, and six primer sets (PCR#3, 4, 6, 7, 9, 10: shown in red) are able to amplify the target sequence when the template genomic DNA contains an inversion of the sacsin-like gene. M1 and M2 indicate the molecular markers, λ-HindIII digest and φX174 HaeIII digest, respectively. PP indicates PCR Products, NC indicates negative control and PC indicates positive control.

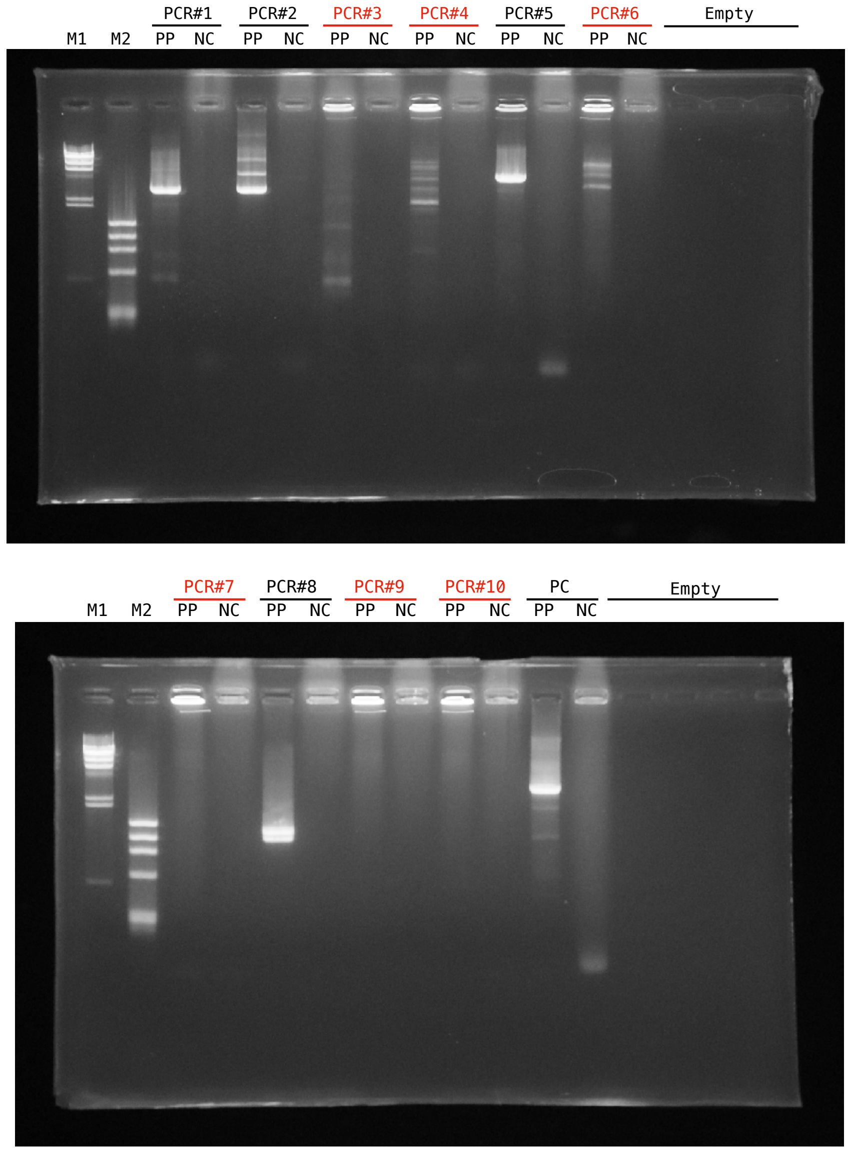

4 Supporting Tables

Table S1. Sample information for high-throughput sequencing data

Sample name	Species	Accession Number
Kerama_Geruma_KrA10	*Acropora digitifera*	DRR099374
Kerama_Geruma_KrA12	*Acropora digitifera*	DRR099376
Kerama_Geruma_KrA13	*Acropora digitifera*	DRR099377
Kerama_Geruma_KrA4	*Acropora digitifera*	DRR099383
Kerama_Geruma_KrA6	*Acropora digitifera*	DRR099385
Kerama_Yakabi_KrC10	*Acropora digitifera*	DRR099359
Kerama_Yakabi_KrC11	*Acropora digitifera*	DRR099360
Kerama_Yakabi_KrC12	*Acropora digitifera*	DRR099361
Kerama_Yakabi_KrC13	*Acropora digitifera*	DRR099362
Kerama_Yakabi_KrC14	*Acropora digitifera*	DRR099363
Kerama_Yakabi_KrC15	*Acropora digitifera*	DRR099364
Kerama_Yakabi_KrC5	*Acropora digitifera*	DRR099369
Kerama_Yakabi_KrC7	*Acropora digitifera*	DRR099371
Kerama_Yakabi_KrC8	*Acropora digitifera*	DRR099372
Kerama_Yakabi_KrC9	*Acropora digitifera*	DRR099373
Kerama_Zamami_KrE1	*Acropora digitifera*	DRR099344
Kerama_Zamami_KrE2	*Acropora digitifera*	DRR099345
Kerama_Zamami_KrE4	*Acropora digitifera*	DRR099347
Kerama_Zamami_KrE5	*Acropora digitifera*	DRR099348
Kerama_Zamami_KrE6	*Acropora digitifera*	DRR099349
Kerama_Zamami_KrE8	*Acropora digitifera*	DRR099351
Okinawa_Manza_Mz1	*Acropora digitifera*	DRR099303
Okinawa_Ohdo_Od10	*Acropora digitifera*	DRR099315
Okinawa_Ohdo_Od11	*Acropora digitifera*	DRR099316

Sample name	Species	Accession Number
Okinawa_Ohdo_Od12	*Acropora digitifera*	DRR099317
Okinawa_Ohdo_Od13	*Acropora digitifera*	DRR099318
Okinawa_Ohdo_Od19	*Acropora digitifera*	DRR099322
Okinawa_Ohdo_Od23	*Acropora digitifera*	DRR099324
Okinawa_Ohdo_ohdo1	*Acropora digitifera*	DRR099334
Okinawa_Ohdo_ohdo3	*Acropora digitifera*	DRR099335
S1601(Adig1)	*Acropora digitifera*	DRR108003
S1603(Adig2)	*Acropora digitifera*	DRR108005
S1606(Adig3)	*Acropora digitifera*	DRR108008
T3(Aten1)	*A. tenuis*	DRR110778
T5(Aten2)	*A. tenuis*	DRR110780
JB05	*A. millepora*	SRR10571416
PA21	*A. millepora*	SRR10571410
FY13	*A. millepora*	SRR10571521
CS11	*A. millepora*	SRR10571429
AN03	*A. millepora*	SRR10571544
TR02	*A. millepora*	SRR10571555
DK04	*A. millepora*	SRR10571373
RB13	*A. millepora*	SRR10571533
FR20	*A. millepora*	SRR10571423
HH16	*A. millepora*	SRR10571477
RL18	*A. millepora*	SRR10571404
NB16	*A. millepora*	SRR10571311
-	*A. millepora*	SRR2086157

Sample name	Species	Accession Number
-	*M. efflorescens*	DRR194233
-	*M. cactus*	DRR194228
-	*M. aequiturberculata*	ERR2190877
-	*M. spumosa*	ERR2282060

Table S2. Primer sequences

Primer name	Primer sequence (5'-3')	Primer position on chr7
R10i1F	CTAGTCATATGGATTGAGACACGATCTTGGRATGCT	13,325,485-13,325,505
R10i2R	ACCCGGGGATCCGATGTTTTCSBTGGAAGAATACTC	13,326,781-13,326,626
Region1_F1	GATAGGAAAACGTCGCTTTCG	13,310,636-13,310,656
Region1_R1	GTATGGATGACTTGCTTATTGC	13,318,404-13,318,425
Region1_F3	GGATSAGACTGAAAGAACCT	13,318,368-13,318,387
Region2_R2	GACCTTGTTTACGAATCTTC	13,334,316-13,334,335
Region1_F2	TGTTTTGGCAAGTTTGACTTG	13,308,830-13,308,850
Region1_R2	TAGAGCTCAATGGACTCGACA	13,311,555-13,311,575
Region2_F2	GAATTRGCSAGAAAGATTGTGGAT	13,331,199-13,331,222
Region1_R4	YAGAACACARGGATGGTGTT	13,319,601-13,319,620

Table S3. PCR analysis (no inversion)

PCR#	Primer name1	Primer name1	Result
#1	Region1_F2	Region1_R2	Amplified
#2	Region2_F2	Region2_R2	Amplified
#5	Region1_F1	Region1_R1	Amplified
#8	Region1_F3	Region1_R4	Amplified

Table S4. PCR analysis (with inversion)

PCR#	Primer name1	Primer name1	Result
#3	Region1_F2	Region2_F2	Many bands
#4	Region1_R2	Region2_R2	Many bands
#6	Region1_F1	Region2_F2	Many bands
#7	Region1_R1	Region2_R2	Not amplified
#9	Region1_F3	Region2_F2	Not amplified
#10	Region1_R4	Region2_R2	Not amplified

Table S5. Information for the isolated nucleotide sequences

Sequence name	Species	The the number of clones determined the sequences
Adig1_seq1	*Acropora digitifera*	3
Adig1_seq2	*Acropora digitifera*	3
Adig2_seq1	*Acropora digitifera*	7
Adig3_seq1	*Acropora digitifera*	4
Adig3_seq2	*Acropora digitifera*	6
Aten1_seq1	*Acropora tenuis*	3
Aten2_seq1	*Acropora tenuis*	6
Aten2_seq2	*Acropora tenuis*	3
Maeq1_seq1	*Montipora aequituberculata*	3
Minf1_seq1	*Montipora informis*	5
Minf1_seq2	*Montipora informis*	14
